# Evaluating the tradeoffs of a generalist parasitoid fungus, *Ophiocordyceps unilateralis*, on different sympatric ant hosts

**DOI:** 10.1038/s41598-020-63400-1

**Published:** 2020-04-14

**Authors:** Wei-Jiun Lin, Yung-I Lee, Shao-Lun Liu, Chung-Chi Lin, Tan-Ya Chung, Jui-Yu Chou

**Affiliations:** 1 Department of Biology, National Changhua University of Education, Changhua, 500 Taiwan; 2 Biology Department, National Museum of Natural Science, Taichung, 404 Taiwan; 3 Department of Life Sciences, National Chung Hsing University, Taichung, 40227 Taiwan; 4 Department of Life Science & Center for Ecology and Environment, Tunghai University, Taichung, 40704 Taiwan

**Keywords:** Microbial ecology, Fungal ecology

## Abstract

It is essential for the survival and reproduction of parasitoids to adapt to the fluctuating host resources. Phenotypic plasticity may enable a parasitoid species to successfully achieve its control over a range of host species to maximize fitness in different hosts that may each require dissimilar, possibly conflicting, specific adaptations. However, there is limited information on how the fitness effects of host switching partition into costs due to the novelty of host species, where unfamiliarity with host physiological and morphological changes and its anti-parasite defenses reduces parasitoid growth, survivorship and/or reproductive success. In this study, the parasitoid fungus *Ophiocordyceps unilateralis sensu lato* was found to sympatrically infect a principal host ant species and other alternative sympatric hosts in the forest of central Taiwan. We herein report that the occurrence of ant infections by *O. unilateralis s.l*. shows spatial and temporal variation patterns on different host species. Results showed that the height from the ground to the leaf where the infected ants grip on, perithecia-forming ability, and growth rate of the stroma of the parasitoid fungus were dissimilar on different host species. These host range expansions not only related the fitness of *O. unilateralis s.l*. but also influenced the expression of extended phenotypic traits. Our findings revealed that a generalist parasitoid fungus suffered an evolutionary tradeoff between host breadth expansion and host-use efficiency.

## Introduction

Parasites (or parasitoids) can indirectly affect the behavior and physiology of their hosts in ways fit only for science fiction, a phenomenon that fascinates both scientists and nonscientists alike. Such behaviors are known as the extended phenotypes of these parasites and are parsimoniously explained as the capacity of parasites to express their genes to modify the host behavior. The parasite manipulation hypothesis suggests that specific behavioral modifications induced in a host can be used by parasites to advance their own fitness^1,2^. One noteworthy example of a parasite’s extended phenotype is the case of the “death grip” of ants infected by entomopathogenic fungal genus *Ophiocordyceps*^3,4^. The parasitic fungus manipulates its ant victims to behave like “zombies,” walking randomly and displaying convulsions that make them fall down, after which the ants climb up vegetation to bite the underside of leaves or twigs^4^. After the ant dies, the fungus continues to grow inside the body, and the fungus’s fruiting body sprouts from the back of the ant’s head. After one or two weeks, the fungus produces spores from the fruiting body and rupture, releasing the spores to the forest floor below. Each spore has the potential to infect other unfortunate ants, turning them into zombie ants that allow the fungus to continue to propagate^3^.

Many biotic and abiotic factors can control or influence which species become hosts to a specific parasite^5–7^. These biotic factors include the variability in the frequency of encounters between the host and the parasite and the ability of the parasite to invade and persist on the host they encounter^8^. Host specificity is one of the most fundamental properties of a parasite, because it can determine whether a parasite can grow and achieve reproductive success. Over the course of evolution, we predict that the natural selection for parasites is to specialize on one host or generalize on a wide range of hosts. Specificity in parasite–host interactions can be influenced by host genotypes that are resistant to only a subset of parasite genotypes and parasite genotypes that are infective on a subset of host genotypes. Parasite–host interaction specificity is driven by the diversity or temporal variability of the host’s population and by genetic trade-offs in adaptation to different hosts. Thus, co-evolutionary consequence critically depends on the pervasiveness of genetic interactions.

*Ophiocordyceps unilateralis* (Clavicipitaceae: Hypocreales), also referred to as a “zombie fungus,” is a fungal pathogen considered specific to ants of the tribe Camponotini (Formicinae: Formicidae). This entomopathogen currently found predominantly in tropical forest ecosystems^9^. Due to the increased amount of research on *Ophiocordyceps* in recent years, the name *O*. *unilateralis* is often extended to *O*. *unilateralis sensu lato*, which suggests that there are several cryptic species within *O*. *unilateralis*, which are yet to be described^9^. Studies have reported that *O*. *unilateralis* in the southern Thailand mainly parasitize carpenter ants, especially *Camponotus leonardi*, as principal hosts with minor supplementary infections found in some *Polyrhachis* ants^3,10^. Evans *et al*.^11^ found diversity within the complex in four different *Camponotus* species in Brazil and suggested that *O*. *unilateralis* seems to be a species complex of widespread distribution with noticeable local diversities of associated hosts. Subsequent papers dealing with new taxa and issues from Thailand, Japan, and Brazilian Amazon on both *Camponotus* and *Polyrhachis* hosts have provided evidence to support for the “one ant, one *Ophiocordyceps* species” hypothesis as raised by Evans *et al*.^11–15^. Thus, *O*. *unilateralis* may have specificity toward different ant species, species delimitation, and host–parasite interaction leading to speciation.

We herein report an *O*. *unilateralis s.l*. species that can sympatrically infect eight ant species, namely *P*. *mesota*, *P*. *wolfi*, *P*. *vigilans*, *P*. *latona*, *P*. *debilis*, *P*. *illaudata*, *P*. *dives*, and *C*. *punctatissimus*, in a broad-leaved forest in central Taiwan. It is very critical to select host species that maximize their fitness for generalist parasitoids. However, evolutionary and ecological determinants underlying alternative host use still remain unclear. In this study, we examined the patterns of alternative host use of this generalist fungus in the wild. To elucidate the evolution of host–parasitoid interactions, understanding the effects of different hosts on the parasitoid is necessary. Altered behaviors that represent the extended phenotypes of different ant hosts due to parasitic manipulation were compared. Furthermore, we measured the parasitoid reproductive success by examining the perithecia-forming ability and growth rate of the stroma of *O*. *unilateralis s.l*. on different ant hosts. Our observations for the first time disobeyed the “one ant, one *Ophiocordyceps* species” hypothesis in this field. Furthermore, this study showed the patterns of alternative host use and unraveled fascinating questions regarding the influence of phenotypic plasticity to the use of a large host spectrum by a generalist parasitoid.

## Materials and Methods

### Study site and ecological monitoring

This study was conducted in the Lienhuachi Experimental Forest (LEF), an evergreen, broad-leaved forest in Nantou County, central Taiwan (23°55'7″N 120°52'58″E). The ecological monitoring of the tempospatial dynamics of ants and their infecting fungi was performed every month from January 2017 to March 2018. The canopy of understory plants with a height lower than approximately 3 m was scanned for dead insects with fungal growth. Specimens were carefully removed by cutting the leaf with the dead ant from the tree and placing it in a 50-mL conical centrifuge tube, which was then transported to the laboratory. LEF is a part of the Taiwan Forest Dynamics Monitoring Network. The substrate of LEF is made of the alternation of argillaceous sandstone and shale. The predominant type of soils in LEF is classified as typical dystrochrept and typical hapludults red soils. The mean annual temperature was 20.8 °C, and the annual precipitation was 2285.0 mm with seasonal variation throughout the year^16^. During ecological monitoring from January 2017 to March 2018, the high-density areas with many dead *O*. *unilateralis s.l*.-infected ants were identified. For all dead infected ants within these areas, we identified the ant based on its morphological features and recorded the height of the dead ant above ground, as well as the position of the dead ant on the leaf. To determine the fitness differences of zombie fungus on different ant hosts, the number of perithecia formed and the length of the stroma in which the perithecia were embedded were also recorded monthly.

### Phylogenetic relationships of zombie-ant fungus

#### DNA extraction

*O*. *unilateralis s.l*.-infected ants were transported to the laboratory and then chilled at 4 °C before performing the following steps. Following the procedure used in our previous study, DNA was extracted from small pieces of the stroma by suspending them in 200 µL of a lysis buffer (2% Triton X-100, 1% sodium dodecyl sulfate, 100 mM sodium chloride, 10 mM Tris [pH 8.0], and 1 mM ethylenediaminetetraacetic acid [EDTA]), to which 200 µL of phenol-chloroform-isoamyl alcohol (25:24:1; isoamyl alcohol is optional) and 0.3 g of acid-washed glass beads (0.45–0.52 mm) were added and gently mixed. The samples were vortexed for 5 min to disrupt the cells and then centrifuged at 13,000–16,000 *g* for 5 min. The aqueous layer of each sample was then transferred to a clean tube, followed by the addition of 400 µL of 95% ethanol and 16 µL of 3 M sodium acetate (pH 5.2). The samples were mixed through inversion and centrifuged at 13,000–16,000 *g* for 5 min. The pellets were then washed with 300 µL of 70% ethanol, and the samples were centrifuged at 13,000–16,000 *g* for 2 min before the supernatant was discarded. Subsequently, the ethanol solution was aspirated with air for 30 min to dry the pellets. Finally, genomic DNA from each sample was suspended in 100 µL of a Tris-EDTA buffer (pH 8.0)^17^.

#### PCR and sequencing

The elongation factor 1α (EF1) and β-tubulin gene regions were amplified via polymerase chain reaction from the genomic DNA extracted from fungal cells. The EF1 gene was partially amplified with the primer 983 F (5′-GCYC CYGGHCAYCGTGAYTTYAT-3′) combined with EFgr (5′-GCAATGTGGGCRGTRTGRCARTC-3), giving a fragment of approximately 800 bp^14,18^. PCR amplification was performed in a thermocycler (G02, ASTEC, Japan) as follows: initial denaturation at 95 °C for 5 min, 35 cycles of denaturation at 95 °C for 1 min, annealing at 50 °C for 30 seconds, and extension at 72 °C for 1 min, followed by a final extension at 72 °C for 5 min. β-tubulin was amplified using the primers T1 (5′-AACATGCGTGAGATTGTAAGT-3′) and T22 (5′-TCTGGATGTTGTTGGGAATCC-3′) spanning a fragment of approximately 1300 bp^19^. For this gene, PCR conditions were as follows: initial denaturation at 95 °C for 5 min, 35 cycles of denaturation at 95 °C for 1 min, annealing at 50 °C for 30 seconds, and extension at 72 °C for 2 min, followed by a final extension at 72 °C for 5 min. PCR reactions for both genes were prepared in 50 µL of solution containing sterile ddH_2_O, 1 X PCR buffer, 200 µM of each dNTP, 0.2 µM of forward and reverse primer, 10~250 ng genomic DNA, and 1.25U Taq polymerase (PU-TQB-500, PURIGO Biotechnology Co., Ltd., Taipei City, Taiwan). The DNA sequencing reactions of these samples was performed at Tri-I Biotech, Inc., New Taipei City, Taiwan.

#### Phylogenetic and network analyses

A list of taxa and fungal samples used in this study, their host identity, and a corresponding NCBI accession number for the EF1 and β-tubulin sequences are presented in Table S1. Multiple alignment was done by using the software MUSHCLE^20^, followed by manually checking the alignment by using BioEdit^21^. Phylogenetic analysis of data was performed using the computer program MEGA version 4. An EF1 sequence of *Ophiocordyceps sinensis* from GenBank (JX968018) was used as the outgroup to the EF1 data and a β-tubulin sequence of *O*. *sinensis* (JX968023) as the outgroup to the β-tubulin data. For the phylogenetic inference, we implemented a maximum-likelihood (ML) analysis by using the program MEGA^22^. Prior to the phylogenetic analysis, the best fit of the nucleotide evolutionary model for each gene was selected based on ML model fitting in the software MEGA. The substitution model for ML was the general-time reversible with gamma-distributed rate heterogeneity (GTR + Γ) as suggested by running “Find best DNA model” implemented in MEGA7. The heuristic search options were conducted using the “level 5” tree bisection and reconnection (TBR). A total of 500 bootstrap replicates for the ML tree topologies were implemented to statistically assess the nodal reliability^23^. Except for phylogenetic analyses, statistical parsimony network analyses were applied to further examine whether or not our samples revealed any population differentiation using the software TCS^24^. The number of substitutions connecting haplotypes in the networks was calculated according to the algorithm described in Templeton *et al*.^25^. In addition to the phylogenetic analysis for each locus alone, we also inferred the phylogenetic relationship of our fungal samples using the concatenated dataset (i.e., the concatenation of the EF1 and β-tubulin sequences). However, the MEGA software does not allow us to perform the evolutionary rate partition of different locus in the concatenated dataset in the phylogenetic inference. We therefore implemented the maximum-likelihood phylogenetic analysis for the concatenated dataset using the online software IQ-TREE with the default settings^26^. The best-fit nucleotide substitution model for both locus were GTR + Γ, consistent with the observations using MEGA. In the IQ-TREE analyses, a total of 1,000 bootstrap replicates analyses was implemented to gain the statistical support in the phylogenetic analysis.

### Species delimitation analyses

To determine the potential cryptic species in *O*. *unilateralis* complex, we applied the algorithm-based species delimitation analyses using the automatic barcode gap discovery (ABGD) approach^27^. The ABGD analyses allowed us to detect the cryptic species in the morphologically undistinguishable group primarily based on a series of prior thresholds (ranging from 0.001 to 0.1) for a gap in the pairwise distribution of genetic distance. The gap is then regarded as a genetic threshold to determine the upper limit of intraspecific distances and the lower limit of interspecific distances. Briefly, the aligned sequence was uploaded to the online ABGD web tool (wwwabi.snv.jussieu.fr/public/abgd/abgdweb.html, date accessed: Feb. 6, 2020) with the default settings. The pairwise genetic distance was estimated using a Kimura 2-parameter model. It has been shown that the recursive partition often overestimated the number of putative species in the ABGD analyses^27^. We therefore only considered the results of initial partition for the inference of putative species number [i.e., operational taxonomic unit (OTU) herein] in this study. We compared the OTU number estimated from each locus alone and the two-loci concatenated dataset, and only took the lowest count as our primary species hypothesis (i.e., the most conservative estimate).

### Ant species identification

Ant identification was carried out based on morphological characters of the family Formicidae with focus on *Camponotus* and *Polyrhachis* species^28–30^. Further characterization was done through amplification and sequencing of the cytochrome c oxidase subunit II gene using the L3034 and H3665A primers as described by Chiotis *et al*.^31^.

### Histological cross sections

Leaf samples were fixed in 1% glutaraldehyde buffered with 0.1 M phosphate buffer, pH 6.8, for 24 hours at room temperature. After fixation, the samples were dehydrated and infiltrated gradually with Technovit 7100 resin (Kulzer GmbH, Germany). The samples were then embedded according to Yeung^32^. Sections, 3-μm thick, were cut using a Ralph knife on a Reichert-Jung 2040 Autocut rotary microtome. Sections were stained with 0.05% (w/v) toluidine blue O (TBO). The sections were viewed, and the images were captured digitally using a CCD camera attached to a light microscope (Axioskop 2, Carl Zeiss AG, Germany).

### Statistical analysis

Unless otherwise stated, statistical analysis was performed using the Data Analysis ToolPak in Microsoft Excel. To examine differences between groups of data, one-way analysis of variance (ANOVA) with least significant difference (LSD) post hoc test was performed. A probability value (*p*) less than 0.05 was considered statistically significant.

## Results

### *O. unilateralis s.l*. can infect eight sympatric ant species

**Figure 1:**
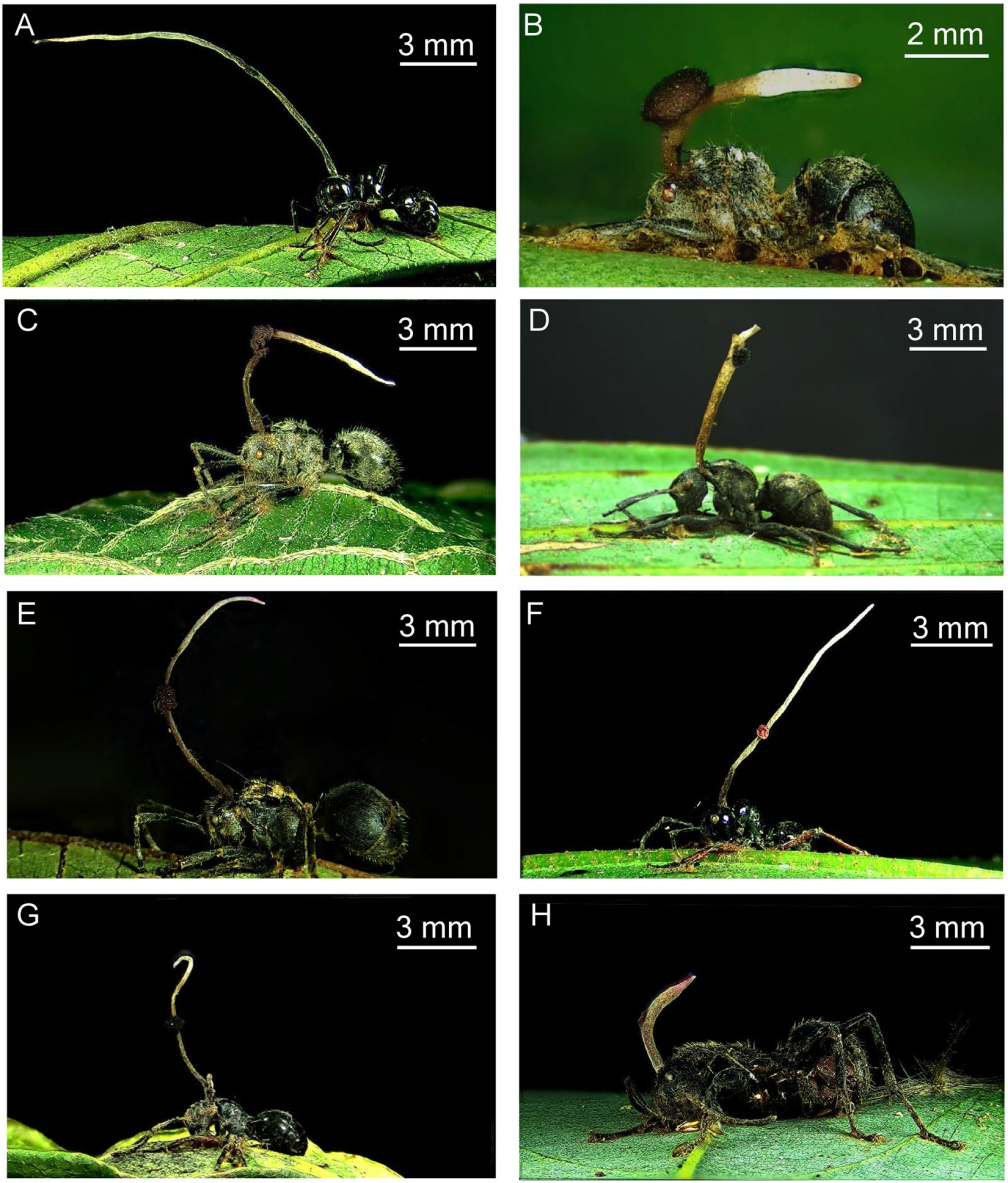
*In situ* photographs showing the infection of *Ophiocordyceps unilateralis sensu lato* on eight different sympatric ant species: *Polyrhachis mesota* (**A**), *Polyrhachis wolfi* (**B**), *Polyrhachis vigilans* (**C**), *Polyrhachis latona* (**D**), *Polyrhachis illaudata* (**E**), *Polyrhachis debilis* (**F**), *Polyrhachis dives* (**G**), and *Camponotus punctatissimus* (**H**).

**Figure 2:**
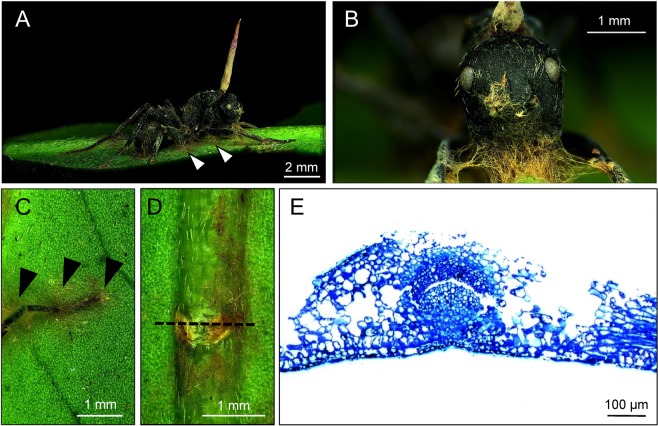
(**A–C**) The secure attachment of the ant to the plants occurs not only through the grip of the ant’s mandibles on major leaf veins along their abaxial surface but also by the fungal rhizoid/mycelial mat fastening the ant’s abdominal segments, mandibles, and legs to the surface of the leaf (arrowheads). (**D**) The eight ant species in this study all express death grip behavior to secure their place, leaving characteristic dumbbell-shaped marks on the plants. (**E**) The histological cross sections (dash lines in **D**) indicated that the fungus does not actually invade the leaf tissue. This shows that the fungus only attaches to the plants.

Based on the morphological traits of ants, the fungus *O*. *unilateralis s.l*. was found on eight ant hosts including *Polyrhachis mesota*, *P*. *wolfi*, *P*. *vigilans*, *P*. *latona*, *P*. *debilis*, *P*. *illaudata*, *P*. *dives*, and *Camponotus punctatissimus* (Fig. 1, Figs. S1–8). The molecular evidence also supports they are eight different ant species (Fig. S9). The stroma arose from the dorsal anterior part of the pronotum. The fertile region of the lateral cushion was hemispherical, dark brown, and further darkened with age. The secure attachment of the ant to the plants occurred not only through the grip of the ant’s mandibles on major leaf veins along their abaxial surface but also by the fungal rhizoid/mycelial mat attaching the ant’s abdominal segments, mandibles, and legs to the underside of a leaf (Fig. 2A–C). It is worth mentioning that the eight ant species all expressed death grip behavior to secure their place, leaving characteristic dumbbell-shaped scars centered around leaf veins (Fig. 2D). This final grip is commonly known as the “death grip” and occurs in very precise locations, most commonly on the underside of leaves. The death grip behavior occurred in all the infected ants. Here, we observed that the locations of host ants on the leaf undersides were highly stable and were always on a major primary vein. Many fungal species are not only opportunistic pathogens in animals but also plant-pathogenic fungi^33–35^. We hypothesize that the vascularized tissue of leaves may provide a nutritional supplement for the developing fungus, because leaf biting may create an opening where fungi gain ready access to plant nutrients. However, the histological cross sections indicated that the fungus does not actually invade the leaf tissue (Fig. 2E); the fungus only attaches to the plants. Therefore, we put forth that the energy for fungal growth might be generated from the infected ants.

### The occurrence of ant infections by *O*. *unilateralis s*.*l*. showed spatial and temporal variation patterns

**Figure 3:**
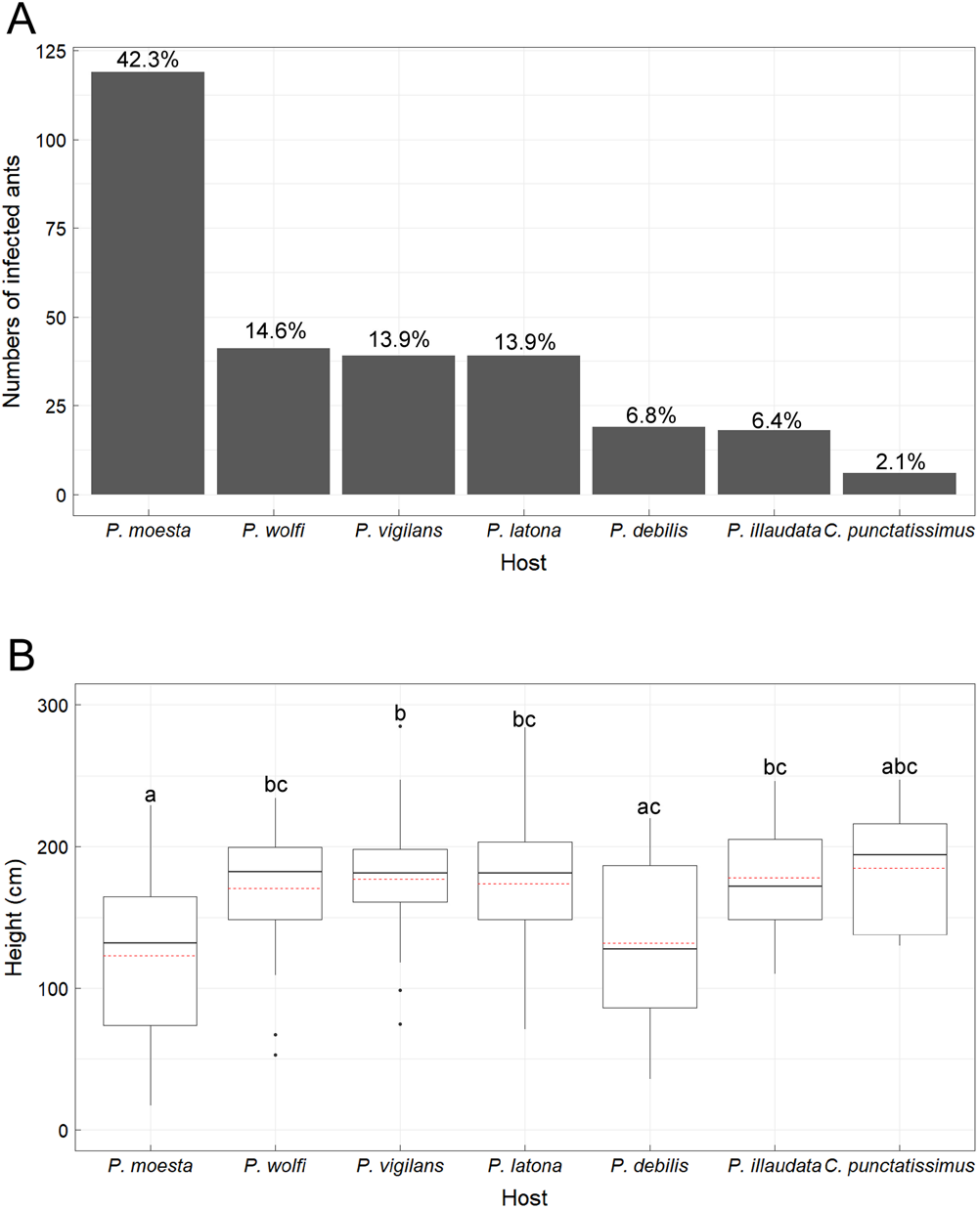
Occurrence of ant infections by *Ophiocordyceps unilateralis sensu lato*. (**A**) Total number of different infected ants from January 2017 to March 2018. **(B)** Height above ground on different infected ants. Data with the same letter are not significantly different from each other according to one-way ANOVA with LSD post hoc test.

Of the 281 infected ants surveyed, 119 were *P*. *mesota* (42.3%), 41 were *P*. *wolfi* (14.6%), 39 were *P*. *vigilans* (13.9%), 39 were *P*. *latona* (13.9%), 19 were *P*. *debilis* (6.8%), 18 were *P*. *illaudata* (6.4%), and 6 were *C*. *punctatissimus* (2.1%) (Fig. 3A). However, very few infected *P*. *dives* were found, and all the samples were used for molecular analysis. Therefore, we excluded these from the following analysis. Thus, we defined *P*. *mesota* as the principal host ant species and others as alternative hosts of *O*. *unilateralis s*.*l*. The infected principal host, *P*. *mesota* ants, were found at a mean height of 123.1 ± 54.3 cm (mean ± SD); 95% confidence interval (CI) = 113.1–133.0 cm; *n* = 114, which was considerably lower than those of the infected alternative hosts, *P*. *wolfi* with a mean height of 170.3 ± 43.2 cm (95% CI = 156.6–184.0 cm; *n* = 38), *P*. *vigilans* with a mean height of 176.9 ± 44.8 cm (95% CI = 162.5–191.3 cm; *n* = 37), *P*. *latona* with a mean height of 173.8 ± 46.1 cm (95% CI = 159.3–188.3 cm; *n* = 39), and *P*. *illaudata* with a mean height of 178.0 ± 39.2 cm (95% CI = 159.4–196.6 cm; *n* = 17), but was not substantially different than those of the other two infected ant species, *P*. *debilis* with a mean height of 131.6 ± 57.3 cm (95% CI = 105.1–158.1 cm; *n* = 18) and *C*. *punctatissimus* with a mean height of 185 ± 50.3 cm (95% CI = 140.9–229.1 cm; *n* = 5). The height of *P*. *debilis*-infected ants was also considerably lower than that of *P*. *vigilans*-infected ants (Fig. 3B)

### Molecular data suggests the *O*. *unilateralis s.l*. studied was a generalist parasitoid

**Figure 4:**
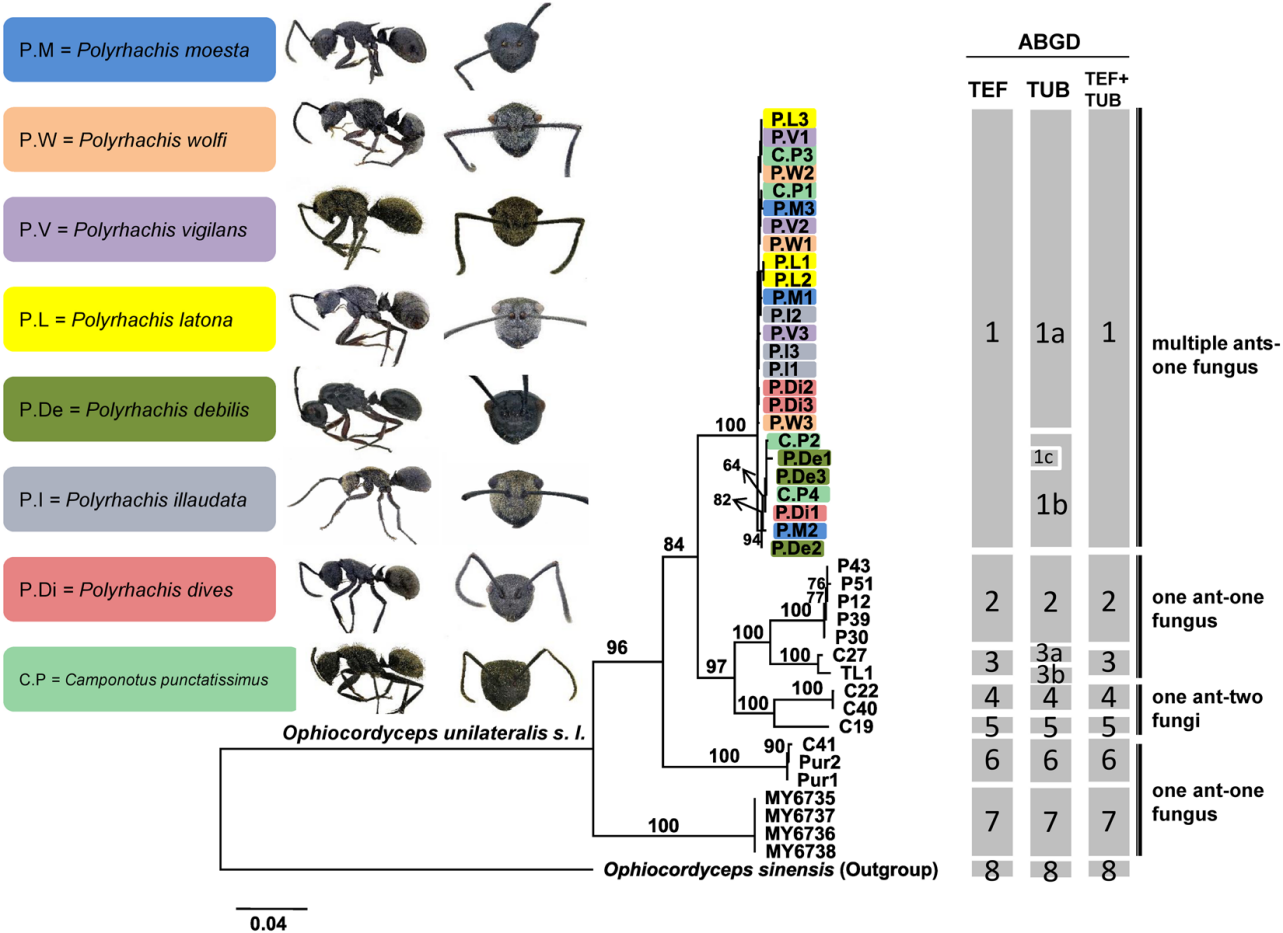
Maximum-likelihood concatenated phylogeny (TUB and EF1) showing the infection of different *Ophiocordyceps unilateralis* samples we collected from eight sympatric ant hosts (marked with color) with the species delimitation results from the ABGD analyses. All collected samples form a monophyletic clade, indicative of the generalized infection of this fungal species. The statistical support (>70%) based on bootstrap analyses are shown above the branches. Scale bar indicates substitutions per site. The columns to the right of the tree indicate the primary species hypothesis (i.e., operational taxonomic unit) estimated based on the ABGD analyses from single locus data (EF1 or β-tubulin) and two-loci concatenated data (EF1 + β-tubulin).

Over the course of our ecological investigations, we randomly examined the identity of infected fungi to determine whether these infectious fungi were of the same or different species. Sequencing results demonstrated that the eight sympatric host ants were largely infected by the same species based on the phylogenetic and network analyses. Prior to the maximum-likelihood phylogenetic analyses, the selection of the best nucleotide substitution model was the general time reversible with heterogeneity between sites (*G* = 0.27) for β-tubulin and the Tamura and Nei 1993 (TN93) model with heterogeneity between sites (*G* = 0.18) for EF1. Similarly, the model selection based on the IQ-TREWEE analyses also yielded the same results as GTR + Γ for both locus in the concatenated dataset. The phylogenetic analysis for each locus alone is largely congruent with the concatenated phylogeny. Thus, we only presented the concatenated phylogeny here (Fig. 4). Our concatenated phylogenetic analyses showed that monophyletic clades were highly supported for both genes alone (Fig. S10) and the concatenated dataset. The ABGD species delimitation analyses on the EF1 showed that seven operational taxonomic units (OTUs) were found in the *O. unilateralis s.l. *(OTU1-7 in Fig. 4). In comparison to EF1, β-tubulin split OTU1 into three taxonomic units and OTU3 into two taxonomic units (Fig. 4). To reconcile the above conflict, we further conducted the ABGD species delimitation analyses using the concatenated dataset. The concatenation of EF1 and β-tubulin showed a total of seven OTUs in *O. unilateralis*. Overall, our ABGD species delimitation analyses using each locus and the concatenated dataset suggested that there are at least seven cryptic species in the *O. unilateralis* *s. l.* in this study. Based on these results, the fungal samples we collected here should be considered as single species (i.e., OTU1) based on the more conservative estimate. It is worth noting that our fungal samples are not the same species analyzed in previous studies considering that the species *O. unilateralis* might harbor more cryptic species than previously appreciated^12–15^. Thus, we propose that *O. unilateralis* samples used herein should be considered as a species complex that requires further taxonomic diagnoses. Next, we examined if the parasitic association between the fungus and ants exhibited any particular population structure. Results from statistical parsimony network analyses showed no discernible population differentiation using both genes (Fig. S11), indicative of a single admixture population of *O. unilateralis* OTU1. Overall, our analyses support the admixture population of *O*. *unilateralis* OTU1, which infected eight ant hosts.

### *O*. *unilateralis* OTU1 occurrence on ants showed correlation with ecological parameters

**Figure 5:**
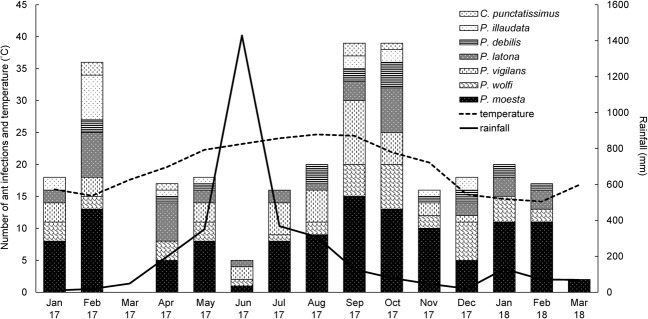
Occurrence of ant infections by *Ophiocordyceps unilateralis* OTU1 from January 2017 to March 2018.

We found a seasonal pattern of the monthly numbers of new dead ants parasited by *O*. *unilateralis* OTU1. One peak of occurrence was found from September to October (Fig. 5). We used data from March 2017 to March 2018 to analyze the correlation between the occurrence of infections and climate factors. It shows no correlation between the number of new dead ants and rainfall found each month (Spearman test: *r* = 0.113, *p* = 0.356). Nevertheless, it seemed that there were substantial increases in the number of new dead ants after high precipitation (Fig. 5). We suggest that it might take time for immature fungal pathogens to reach maturity and for mature pathogens to become revitalized. Thus, it is very possible that a time lag between the precipitation and a peak in the number of new dead ants. We reanalyzed our data to consider a potential lag time of one, two, or three months. There was a significant correlation between the number of new dead ants and the amount of rainfall after a three-month time lag (Spearman test: *r* = 0. 543, *p* < 0.05), whereas no significant correlation was found after a two-month time lag (Spearman test: *r* = 0.373, *p* = 0.104) and either a one-month time lag (Spearman test: *r* = 0.015, *p* = 0.479) based on this reanalysis. Interestingly, we also found that there was a significant difference in temperature between the rainy season (from April to September, 2017; 23.06 ± 1.75 °C) and the dry season (from January to March, 2017 and from October 2017 to March 2018; 16.88 ± 2.50 °C; *t*-test, *p* < 0.001). It indicated that no significant correlation between the number of new dead ants and the temperature when omitting the time lag (Spearman test: *r* = 0.232, *p* = 0.222) or using a three-month time lag (Spearman test: *r* = 0.444, *p* = 0.07), but a two-month (Spearman test: r = 0.57, p < 0.05) and one-month (Spearman test: *r* = 0.484, *p* < 0.05) time lag resulted in a significant correlation between these parameters.

### Reproductive fitness of *O*. *unilateralis* OTU1 differed between ant hosts

**Figure 6:**
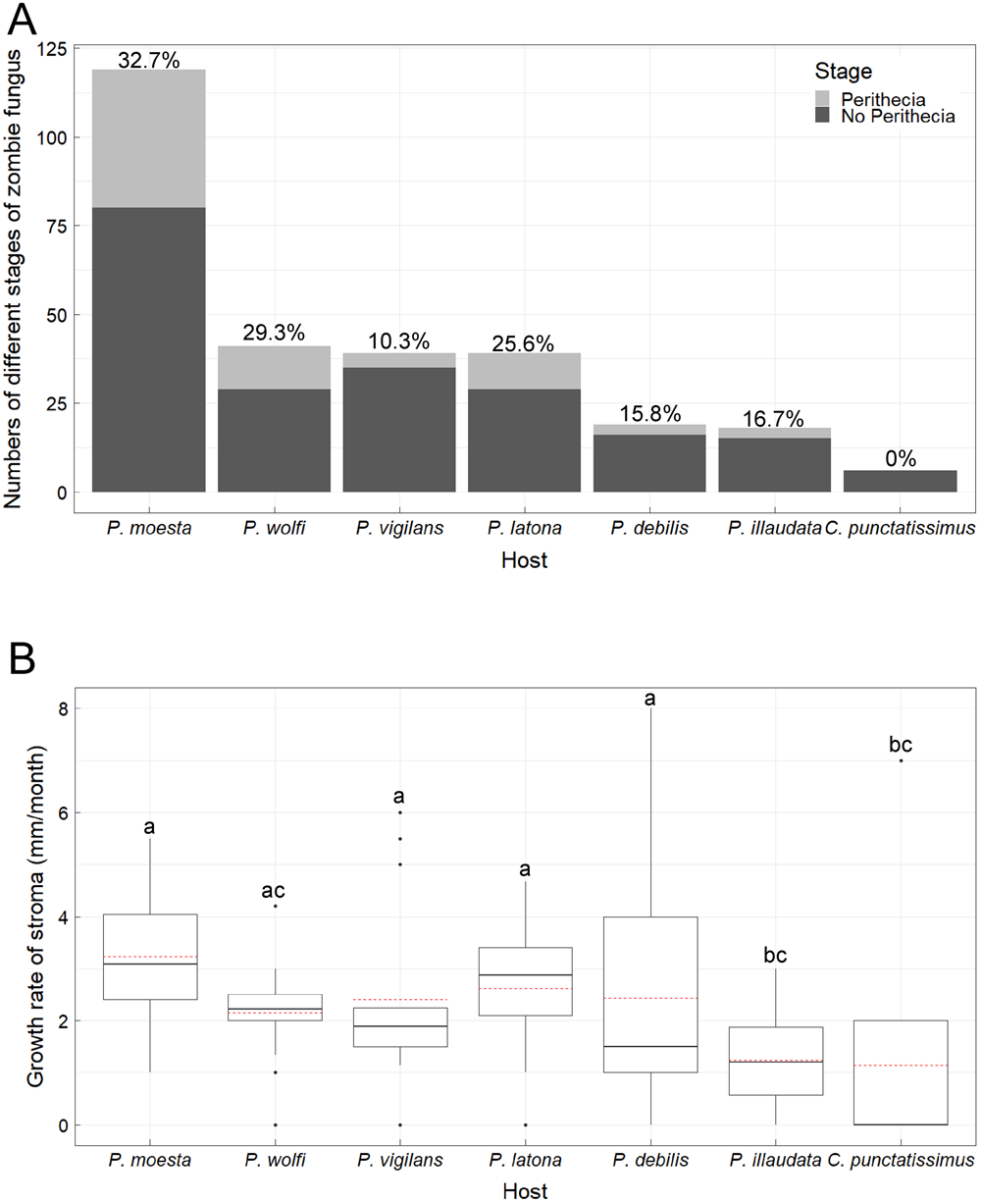
Fitness differences of *Ophiocordyceps unilateralis* OTU1 on different infected ant hosts (**A**) the ratio of perithecia formation. (**B**) the average growth rate of stroma. Data with the same letter are not significantly different from each other according to one-way ANOVA with LSD post hoc test.

The fungal growth cycle after the ant’s death comprised of three main stages: the dead ant stage, the stroma stage, and the perithecia stage. The perithecia develops on the *O*. *unilateralis* OTU1 stroma protruding from the ant’s head and subsequently releases sexual spores. To improve our understanding of the ecology of zombie fungus, we assessed the fitness-related traits of *O*. *unilateralis* OTU1 on different ant hosts. These included perithecia formation on the stroma and the growth rate of the stroma on different infected ant species. In our approximately one-year survey in the field, we found the percentage of perithecia formation was different on different ant hosts. Of the 281 infected ants surveyed, the percentage of perithecia formation was 32.7% on *P*. *mesota*, 29.3% on *P*. *wolfi*, 10.3% on *P*. *vigilans*, 25.6% on *P*. *latona*, 15.8% on *P*. *debilis*, 16.7% on *P*. *illaudata*, and 0% on *C*. *punctatissimus* (Fig. 6A). The effects of host cells on stromal growth were also studied. The growth rate of the stroma was 32.30 ± 12.29 (mean ± SD) mm/month on *P*. *mesota*, 21.45 ± 9.67 mm/month on *P*. *wolfi*, 24.13 ± 17.74 mm/month on *P*. *vigilans*, 26.15 ± 12.56 mm/month on *P*. *latona*, 24.29 ± 24.09 mm/month on *P*. *debilis*, 12.43 ± 8.97 mm/month on *P*. *illaudata*, and 11.43 ± 19.16 mm/month on *C*. *punctatissimus* (Fig. 6B). The growth rate of the stroma on *P*. *mesota* was substantially faster than that on *P*. *illaudata* and *C*. *punctatissimus*. However, we did not find notable differences in the growth rate of the stroma between the principal host, *Polyrhachis mesota*, and the other four alternative hosts, *P*. *wolfi*, *P*. *vigilans*, *P*. *latona*, and *P*. *debilis*. The variance in the growth rates of the stroma on these four hosts was much wider than that on *Polyrhachis mesota*, which is indicative of developmental instability in these stroma. In summary, we found that the fitness of *O. unilateralis* OTU1 on alternative host species was lower than that on the principal host species, suggesting that a generalist parasitoid fungus suffers a trade-off between host breadth expansion and host-use efficiency.

## Discussion

In this study, the results of our molecular phylogenetic analyses revealed that a single species of *O*. *unilateralis* (i.e. OTU1) could infect at least eight different ant species in the forest of central Taiwan. In contrast to the findings of previous studies^14,15^, we did not find any evidence of cryptic parasite genetic structure, strongly arguing against the evolution of host specificity for the fungal pathogen have been examined. Considering that the *O*. *unilateralis* OTU1 we collected is a single species, but can infected eight sympatric ant species. The conflicted observations between our work and previous works can possibly reflect the differences of population dynamics of ant hosts among different forests and is an important issue for future investigation.

Understanding evolutionary forces that act on host specialization has been a question of long-standing interest to evolutionary ecologists^36,37^. A previous study showed that the degree of host specialization may vary geographically or temporally and may also be limited by the phylogenetic relatedness of potential host species^38^. Host specialization may be more effective in source use and is a critical ecological characteristic of parasitic species. However, the parasitic strategy of the zombie fungus, the *O*. *unilateralis* complex, is ambiguous. The *O*. *unilateralis* complex is a ubiquitous pathogen of ants with hidden phylogenetic diversity associated with host specificity. Kobmoo *et al*.^14^ indicated that the *O*. *unilateralis* complex was found to occur predominantly on three ant species, namely *C*. *leonardi*, *C*. *saundersi*, and *P*. *furcate*, in Thailand. In addition, Kobmoo and colleagues indicated that there is a separation of the *O*. *unilateralis* samples into three clades based on molecular data, reflecting specificity to each of the three different hosts ant species. Araújo *et al*. described three new and host-specific species of the *O*. *unilateralis* complex on *Camponotus* ants from the central Amazonian region of Brazil by morphological traits, behavior of ascospores, and DNA sequence data. Most of these studies, if not all, further strengthened the hypothesis that the populations of *O*. *unilateralis s.l*. diverged with their different hosts through host specificity^15^. Here, we however showed that a single *O*. *unilateralis* species could sympatrically infect at least eight ant species (belonging to two genera) in a broad-leaved forest in central Taiwan. Thus, highly specialized and super-generalistic species both exist in this species complex. From the evolutionary perspective, a classic question has been: Why should a parasitic fungus be either host generalist or specialist? For example, similar to our observations here and those reported in the previous literature, the patterns of alternative host use for *O*. *unilateralis* complex mostly vary over spatial scales. In this species complex, it is yet to be clarified why some species can be highly specific in a given habitat and highly generalist in another habitat.

Host ecology and physiology act as “filters” affecting specificity and predominate in parasites that interact minimally with their hosts. The host–parasite interactions depend on two crucial steps: a compatibility filter (i.e., the immunological and physiological compatibility between hosts and parasites) and an encounter filter (i.e., the probability of encounter between hosts and parasites)^6,39^. Depending on the result of interactions between host factors and environmental factors, there are both pros and cons for either specialization or generalization. Parasites that are capable of invading a wide variety of host species might hedge their bets against the risk of extinction by reducing their dependence on a single resource^40^. Concerning the characteristics of the alternative host species being used by a generalist parasite, a second key question is raised: What causes an alternative host more appropriate than another one? A parasite is more likely to use alternative host species that are phylogenetically related and ecologically similar to the principal host species^41^. These two factors are likely to interact because the phylogenetically closely related species often have similar ecological preference^42,43^. This might explain why we found a single *O*. *unilateralis s*.*l*. species that can naturally infect seven sympatric *Polyrhachis* ant hosts. However, how do specialized parasites otherwise shift to a relatively novel host (such as from *Polyrhachis* to *Camponotus* or *vice versa* herein)? Many studies have shown that many groups exhibit a high evolutionary transition rate from generalization to specialization, but the opposite direction is much rarer. However, some studies have suggested that host specialization shows to be dynamic with no inherent necessary directionality^44,45^. Specialization has often been suggested to be an evolutionary dead end, thereby reinforcing specialists to selectively evolve to generalists. However, this is what we called a “parasite paradox”: How do highly specialized parasites otherwise shift to novel hosts? The parasite should retain the ability to use both the ancestral and novel host if they colonize to a novel host. However, extra hosts should typically be inferior alternatives to the original host, to which is specifically adapted by the parasite.

There are some ways in which specialized parasites colonize shift to novel hosts. First, phenotypic plasticity can allow the parasite to generate a strategy infecting new hosts^46^. Second, a correlated trait evolution can generate new traits that are prepared to infect some potential hosts^47^. Third, the conservation of phylogenetic relatedness is related to resource allocation, including biological constraints and reservation of traits from selection pressures before^48,49^. Thus, this suggests that host shifts are often launched because the parasite is exapted (or preadapted) to the novel resource. In other words, the novel host might share critical traits with the current host or might have been used in the past^37^. These pressures provide potential ability to infect new hosts with defense systems are almost identical or sufficiently similar to those of ancestral hosts^50^. This may be evident to explain how specialized parasites otherwise shift to related novel hosts. Host shifts initiated by related novel hosts are not a terminal point but rather one step in the process that accelerates biological expansion and generates novel combinations of interacting species. Previous studies have proposed that parasites’ diversification is influenced by repeated “oscillations” in the host range^51–53^. The oscillation hypothesis depends on the continuous regeneration of changes in host use. The host range expansion during geographic range and environmental expansion followed by phases of host specialization during sympatric speciation or geographic isolation by host race formation. Such a diversity of hosts would spur the diversification of their associated parasites, whether the actual processes occur during speciation relies on sympatric or allopatric modes^52^. The generalist species may become more diverse with preferential production of specialized descendants. The selection pressures promote generalist species to become specific. Based on some gene flow barriers, these generalist species may separate through rapid evolutionary changes in small populations and reinforcement of coadapted gene complexes^54,55^ or through changes in phenology that become associated with mating patterns^56,57^. After divergent local adaptation, these new interactions can facilitate evolutionary diversity^51,58^.

The intensity of selection for resistance traits in the host can be influenced by the spatiotemporal variation in encounter rates with a parasite. The eight host species we investigated here, although sympatric, are found to utilize habitat in dissimilar ways. The nesting and socioecological habits of these species are also different^59,60^. In addition, the occurrence of ant infections by *O. unilateralis s.l*. showed a temporal variation pattern on different host species. This suggests that *O*. *unilateralis*. *s.l*. infected different ant hosts to keep the infection rate to a certain extent throughout the whole year. This phenomenon allowed the parasite to achieve stable infections every month. Here, we found a unique peak of fungal infection during the summer season and a similar observation was made by Pontoppidan *et al*.^10^. However, two peaks of fungal infections were found by Mongkolsamrit *et al*.^61^ they are in the middle of the rainy season and dry season, respectively. The presence or absence of parasites within host populations might not only rely on both biotic but also abiotic factors. The number of new dead ants correlates with the amount of rainfall when a time lag of three months is included. But there is no correlation when no time or one to two months’ lag is considered. It may reflect the fact that this entomogenous fungus takes a certain time to grow, develop and to infect the ant hosts. This suggests that the heavy precipitation of the rainy season stimulates fungal development and can explain the peak pattern we indicated above. Furthermore, the average temperature also varied between months and was correlated with fungal infection when a one- or two-month time lag was considered. Thus, climatic factors, such as rainfall and temperature, probably both play roles in the population dynamics of *O*. *unilateralis s*.*l*.

Here, we report that the height from the ground to the leaf where the infected ant gripped on, perithecia-forming ability, and growth rate of the stroma of the parasitoid fungus varies in a host-dependent fashion. However, the death-grip behavior could be found in all the principal host ant species and a series of alternative sympatric hosts even when they belonged to different genera. These characteristic bite marks have been found on 48-million-year-old fossilized leaves^62^. During the fungal infection progress, once the ant mandibles are secured to the leaf vein, atrophy immediately sets in. Then, the sarcomere connections in the muscle fibers are destroyed and the sarcoplasmic reticula and mitochondria are reduced. After that, the ant is not able to control the muscles of the mandible and will keep fixed in place. This lockjaw trait or death grip is essential in the fungus’s lifecycle. Of note, ants infected by *O*. *unilateralis* complex are predominantly manipulated to bite leaves in tropical forests^3,15,63^. By contrast, fungal development in temperate forests is longer than the period of time leaves are present and the ants are manipulated to bite twigs^64,65^. Loreto *et al*. used an ancestral state reconstruction method to suggest that the leaf biting was likely the ancestral trait and that the twig biting was likely a convergent trait in global temperate regions^66^. The remarkable thing is that the death grip occurs at a very precise location (at the proper height above the forest floor) and is essential to ensure an optimum microclimatic site for the next steps of fungal development and spore release^3^. This behavior is highly conserved, and some entomopathogenic fungi that are able to colonize plant tissues as symptomless endophytes are now evident^67,68^. Thus, leaf biting may also create an opening on the leaf where fungi can gain ready access to plant nutrients^66^. However, our experimental data do not support this hypothesis. We found that the fungal hyphae invaded into the dead body of the ant and expanded onto the surface of the leaf without invading the mesophyll of the leaf. In our previous study, we reported that another parasitic *Ophiocordyceps* species, *O*. *pseudolloydii*, could naturally infect *Dolichoderus thoracicus* ant hosts^69^. Notably, we found that the ant’s mandible grip on the vein or leaf edge did not appear to anchor it in place, as we found on *O*. *unilateralis s*.*l*.-infected *Polyrhachis* and *Camponotus* ants here. However, the dense matrix of the fungal hyphae that sprouted from the ant securely anchored it to the plant. Similar to the results of this study, the histological cross sections showed that the fungus did not actually intrude the leaf tissue and the fungal hyphae only attached to the plants. Therefore, nutrition for fungal growth mostly relies on the infected ants.

In conclusion, our results show a clear difference in the host specificity of *O*. *unilateralis s*.*l*. between the populations in Taiwan and other countries. We found no evidence for cryptic genetic structure in this *O*. *unilateralis* species (OTU1) that could infect multiple sympatric host species; rather, *O*. *unilateralis s*.*l*. was completely panmictic across hosts with high genetic diversity within host populations. Our data also provides evidence for the hypothesis that the generalist’s foraging strategy may result in behavioral as well as physiological trade-offs in the processes of consuming alternative hosts. In other words, a generalist is a “jack of all trades, master of none.” Our explanations for the existence of specialists in this species complex invoke a generalist–specialist fitness trade-off as we have demonstrated in this study. The benefit of being able to do many things comes at the cost of being unable to do those things well.

## Supplementary information

Supplementary information.

## References

[1] Moore J, Adamo S, Thomas F (2005). Manipulation: expansion of the paradigm. Behav. Processes.

[2] Dawkins, R. The extended phenotype: the long reach of the gene. *Oxford University Press*, *Oxford* (1982).

[3] Andersen SB (2009). The life of a dead ant: the expression of an adaptive extended phenotype. Am. Nat..

[4] Hughes DP (2011). Behavioral mechanisms and morphological symptoms of zombie ants dying from fungal infection. BMC Ecol..

[5] Berger V, Galaktionov KV, Prokof’ev VV (2001). Effect of parasites on host adaptation to abiotic environmental factors: host-parasite relationship of trematode parthenites–mollusc system. Parazitologiia.

[6] Poulin, R. Evolutionary ecology of parasites. *Princeton University Press*, *Princeton* (2011).

[7] Schmid-Hempel, P. Evolutionary parasitology the integrated study of infections, immunology, ecology, and genetics. *Oxford University Press*, *Oxford* (2011)

[8] Combes, C. Parasitism: the ecology and evolution of intimate interactions. *University of Chicago Press*, *Chicago* (2001)

[9] Evans HC, Elliot SL, Hughes DP (2011). Ophiocordyceps unilateralis: A keystone species for unraveling ecosystem functioning and biodiversity of fungi in tropical forests?. Commun. Integr. Biol..

[10] Pontoppidan MB, Himaman W, Hywel-Jones NL, Boomsma JJ, Hughes DP (2009). Graveyards on the move: the spatio-temporal distribution of dead ophiocordyceps-infected ants. Plos One.

[11] Evans HC, Elliot SL, Hughes DP (2011). Hidden diversity behind the zombie-ant fungus Ophiocordyceps unilateralis: four new species described from carpenter ants in Minas Gerais, Brazil. Plos One.

[12] Luangsa-Ard JJ, Ridkaew R, Tasanathai K, Thanakitpipattana D, Hywel-Jones N (2011). Ophiocordyceps halabalaensis: a new species of Ophiocordyceps pathogenic to Camponotus gigas in Hala Bala Wildlife Sanctuary, Southern Thailand. Fungal. Biol..

[13] Kepler RM, Kaitsu Y, Tanaka E, Shimano S, Spatafora JW (2011). Ophiocordyceps pulvinata sp. nov., a pathogen of ants with a reduced stroma. Mycoscience.

[14] Kobmoo N, Mongkolsamrit S, Tasanathai K, Thanakitpipattana D, Luangsa-Ard JJ (2012). Molecular phylogenies reveal host-specific divergence of Ophiocordyceps unilateralis sensu lato following its host ants. Mol. Ecol..

[15] Araújo, J. P. M., Evans, H. C., Geiser, D. M., Mackay, W. P. & Hughes, D. P. Unravelling the diversity behind the *Ophiocordyceps unilateralis* (Ophiocordycipitaceae) complex: Three new species of zombie-ant fungi from the Brazilian Amazon. *Phytotaxa* **220**, 224–238 (2015).

[16] Chang L-W (2010). Species composition, size-class structure and diversity of the Lienhuachih forest dynamics plot in a subtropical evergreen broad-leaved forest in central Taiwan. Taiwan J. For. Sci..

[17] Sun, P.-F., Chen, P.-H. & Lin, W.-J. Lin, C.-C. & Chou, J.-Y. Variation in the ability of fungi in the extrafloral nectar of *Mallotus paniculatus* to attract ants as plant defenders. *Mycosphere* **9**, 178–188 (2018).

[18] Rehner, S. A. & Buckley, E. A *Beauveria* phylogeny inferred from nuclear ITS and EF1-alpha sequences: evidence for cryptic diversification and links to *Cordyceps* teleomorphs. *Mycologia* **97**, 84–98 (2005). 10.3852/mycologia.97.1.84 16389960

[19] Glass NL, Donaldson GC (1995). Development of primer sets designed for use with the PCR to amplify conserved genes from filamentous ascomycetes. Appl. Environ. Microbiol..

[20] Edgar RC (2004). MUSCLE: a multiple sequence alignment method with reduced time and space complexity. BMC Bioinformatics.

[21] Hall TA (1999). BioEdit: a user-friendly biological sequence alignment editor and analysis program for Window 95/98/NT. Nucleic Acids Symp. Ser..

[22] Kumar S, Stecher G, Tamura K (2016). MEGA7: molecular evolutionary genetics analysis version 7.0 for bigger datasets. Mol. Biol. Evol..

[23] Felsenstein J (1985). Confidence limits on phylogenies: an approach using the bootstrap. Evolution.

[24] Clement M, Posada D, Crandall KA (2000). TCS: a computer program to estimate gene genealogies. Mol. Ecol..

[25] Templeton AR, Crandall KA, Sing CF (1992). A cladistic analysis of phenotypic associations with haplotypes inferred from restriction endonuclease mapping and DNA sequence data. III. Cladogram estimation. Genetics.

[26] Nguyen LT, Schmidt HA, Von Haeseler A, Minh BQ (2015). IQ-TREE: a fast and effective stochastic algorithm for estimating maximum-likelihood phylogenies. Mol. Biol. Evol..

[27] Puillandre N, Lambert A, Brouillet S, Achaz G (2012). ABGD, Automatic Barcode Gap Discovery for primary species delimitation. Mol. Ecol..

[28] Lin C-C, Wu W-J (2003). The ant fauna of Taiwan (Hymenoptera: Formicidae), with the keys to subfamilies and genera. Annu. Natl. Taiwan Mus..

[29] Terayama M (2009). A synopsis of the family Formicidae of Taiwan (Insecta: Hymenoptera). Liberal Arts, Bull. Kanto Gakuen Univ..

[30] Leong, C.-M., Hsiao, Y. & Shiao, S.-F. *Polyrhachis* (*Cyrtomyrma*) *debilis* Emery, 1887 (Hymenoptera: Formicidae), a New Record of Ant Species in Taiwan. *Formosan Entomologist* **35**, 143–147 (2015).

[31] Chiotis M, Jermiin LS, Crozier RH (2000). A molecular framework for the phylogeny of the ant subfamily Dolichoderinae. Mol. Phylogenet. Evol..

[32] Yeung EC (1999). The use of histology in the study of plant tissue culture systems—some practical comments. In Vitro Cell. Dev..

[33] Zhang N (2006). Members of the Fusarium solani species complex that cause infections in both humans and plants are common in the environment. J. Clin. Microbiol..

[34] Pal M, Dave P, Manna AK (2014). Emerging role of Aspergillus flavus in human and animal disorders. J. Mycopathol. Res..

[35] Srinivas, C. *et al*. *Fusarium oxysporum* f. sp. *lycopersici* causal agent of vascular wilt disease of tomato: Biology to diversity–A review. *Saudi J. Biol. Sci.* **26**, 1315–1324 (2019). 10.1016/j.sjbs.2019.06.002 PMC6864208 31762590

[36] Futuyma DJ, Moreno G (1988). The evolution of ecological specialization. Annu. Rev. Ecol. Evol. Syst..

[37] Agosta SJ, Janz N, Brooks DR (2010). How specialists can be generalists: resolving the” parasite paradox” and implications for emerging infectious disease. Zoologia (Curitiba).

[38] Fallon SM, Bermingham E, Ricklefs RE (2005). Host specialization and geographic localization of avian malaria parasites: a regional analysis in the Lesser Antilles. Am. Nat..

[39] Combes C (1991). Ethological aspects of parasite transmission. Am. Nat..

[40] Bush AO, Kennedy CR (1994). Host fragmentation and helminth parasites: hedging your bets against extinction. Int. J. Parasitol..

[41] Cooper N (2012). Phylogenetic host specificity and understanding parasite sharing in primates. Ecol. Lett..

[42] Burns JH, Strauss SY (2011). More closely related species are more ecologically similar in an experimental test. Proc. Natl. Acad. Sci. USA.

[43] Huang S, Bininda-Emonds OR, Stephens PR, Gittleman JL, Altizer S (2014). Phylogenetically related and ecologically similar carnivores harbour similar parasite assemblages. J. Anim. Ecol..

[44] Nosil P, Mooers A (2005). Testing hypotheses about ecological specialization using phylogenetic trees. Evolution.

[45] Janz N, Nyblom K, Nylin S (2001). Evolutionary dynamics of host-plant specialization: a case study of the tribe Nymphalini. Evolution.

[46] West-Eberhard, M. J. Developmental plasticity and evolution. *Oxford University Press*, *Oxford* (2003).

[47] Lande R, Arnold SJ (1983). The measurement of selection on correlated characters. Evolution.

[48] Gould SJ, Lewontin RC (1979). The spandrels of San Marco and the Panglossian paradigm: a critique of the adaptationist programme. Proc. R. Soc. B.

[49] Janzen DH, Martin PS (1982). Neotropical anachronisms: the fruits the gomphotheres ate. Science.

[50] McLennan DA, Brooks DR (2002). Complex histories of speciation and dispersal in communities: a re-analysis of some Australian bird data using BPA. J. Biogeogr..

[51] Janz N, Nylin S, Wahlberg N (2006). Diversity begets diversity: host expansions and the diversification of plant-feeding insects. BMC Evol. Biol..

[52] Janz, N. & Nylin, S. The oscillation hypothesis of host-plant range and speciation. In: Tilmon, K. (ed.) Specialization, speciation, and radiation: the evolutionary biology of herbivorous insects. *University of California Press*, *Oakland*, pp 203–215 (2008).

[53] Nylin S, Janz N (2009). Butterfly host plant range: an example of plasticity as a promoter of speciation?. Evol. Ecol..

[54] Mayr, E. Animal species and evolution. *Harvard**University**Press*, *Cambridge* (1963).

[55] Thompson, J. N. The coevolutionary process. *University**of Chicago Press*, Chicago (1994).

[56] Bush GL (1994). Sympatric speciation in animals: new wine in old bottles. Trends Ecol. Evol..

[57] Berlocher SH, Feder JL (2002). Sympatric speciation in phytophagous insects: moving beyond controversy?. Annu. Rev. Entomol..

[58] Agosta SJ, Klemens JA (2008). Ecological fitting by phenotypically flexible genotypes: implications for species associations, community assembly and evolution. Ecol. Lett..

[59] Robson SK, Kohout RJ (2007). A review of the nesting habits and socioecology of the ant genus Polyrhachis Fr. Smith. Asian Myrmecol..

[60] Severinghaus LL (2007). Cavity dynamics and breeding success of the Lanyu Scops Owl (Otus elegans). J. Ornithol..

[61] Mongkolsamrit, S. *et al*. Life cycle, host range and temporal variation of *Ophiocordyceps unilateralis*/*Hirsutella formicarum* on Formicine ants. *J. Invertebr. Pathol.* **111**, 217–224 (2012). 10.1016/j.jip.2012.08.007 22959811

[62] Hughes DP, Wappler T, Labandeira CC (2011). Ancient death-grip leaf scars reveal ant-fungal parasitism. Biol. Lett..

[63] Loreto RG, Elliot SL, Freitas ML, Pereira TM, Hughes DP (2014). Long-term disease dynamics for a specialized parasite of ant societies: a field study. Plos One.

[64] Kepler, R. M., Kaitsu, Y., Tanaka, E., Shimano, S. & Spatafora, J. W. *Ophiocordyceps pulvinata* sp. nov., a pathogen with a reduced stroma. *Mycoscience* **52**, 39–47 (2011).

[65] De Bekker C (2014). Species-specific ant brain manipulation by a specialized fungal parasite. BMC Evol. Biol..

[66] Loreto RG (2018). Evidence for convergent evolution of host parasitic manipulation in response to environmental conditions. Evolution.

[67] Vega FE (2008). Insect pathology and fungal endophytes. J. Invertebr. Pathol..

[68] Vidal S, Jaber LR (2015). Entomopathogenic fungi as endophytes: plant–endophyte–herbivore interactions and prospects for use in biological control. Curr. Sci..

[69] Chung T-Y (2017). Zombie ant heads are oriented relative to solar cues. Fungal. Ecol..

