# Supplementary material for: Evaluating the tradeoffs of a generalist parasitoid fungus, Ophiocordyceps unilateralis, on different sympatric ant hosts
Source: Sci Rep. 2020 Apr 14;10:6428. doi: 10.1038/s41598-020-63400-1 (PMC7156370; doi:10.1038/s41598-020-63400-1)
Supplement: Supplementary file 1 — Supplementary information. [file 41598_2020_63400_MOESM1_ESM.pdf]

Evaluating the tradeoffs of a generalist parasitoid fungus, *Ophiocordyceps unilateralis*, on different sympatric ant hosts

Wei-Jiun Lin[1], Yung-I Lee[2,3], Shao-Lun Liu[4], Chung-Chi Lin[1], Tan-Ya Chung[1], and Jui-Yu Chou[1]*

[1]Department of Biology, National Changhua University of Education, Changhua 500, Taiwan

[2]Biology Department, National Museum of Natural Science, Taichung 404, Taiwan

[3]Department of Life Sciences, National Chung Hsing University, Taichung 40227, Taiwan

[4]Department of Life Science & Center for Ecology and Environment, Tunghai University, Taichung 40704, Taiwan

*Corresponding author. Tel: +886-4-7232105 Ext. 3426; Fax: 886-4-7211156; Email: jackyjau@cc.ncue.edu.tw

Supplementary Figures

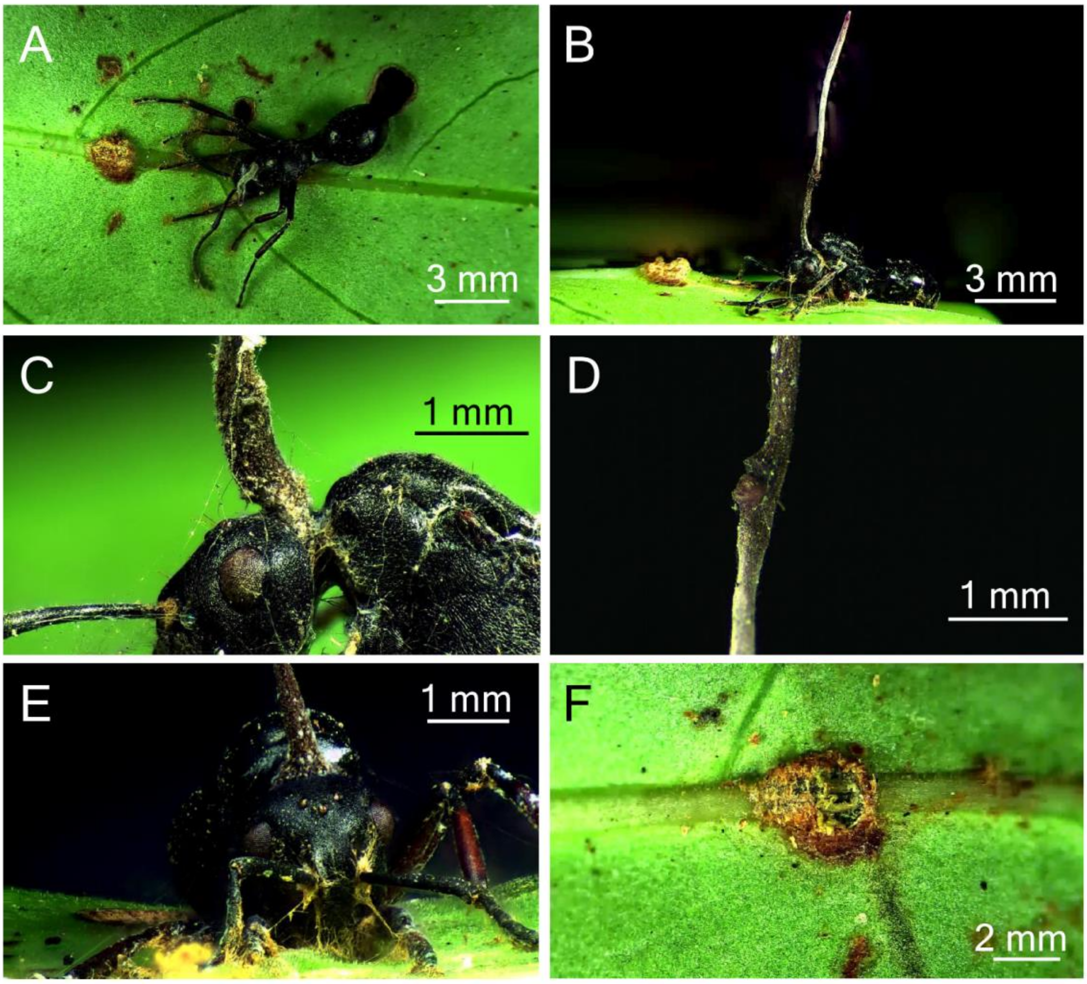

Supplementary Fig. 1 *Ophiocordyceps unilateralis sensu lato*-infected *Polyrhachis mesota* (A) top view (B) side view (C) emerging stroma from the dorsal neck (D) stromata containing immature perithecia (E) frontal view of the ant biting the vein of the leaf (F) scars centered around its veins after the ant was gently removed from the leaf.

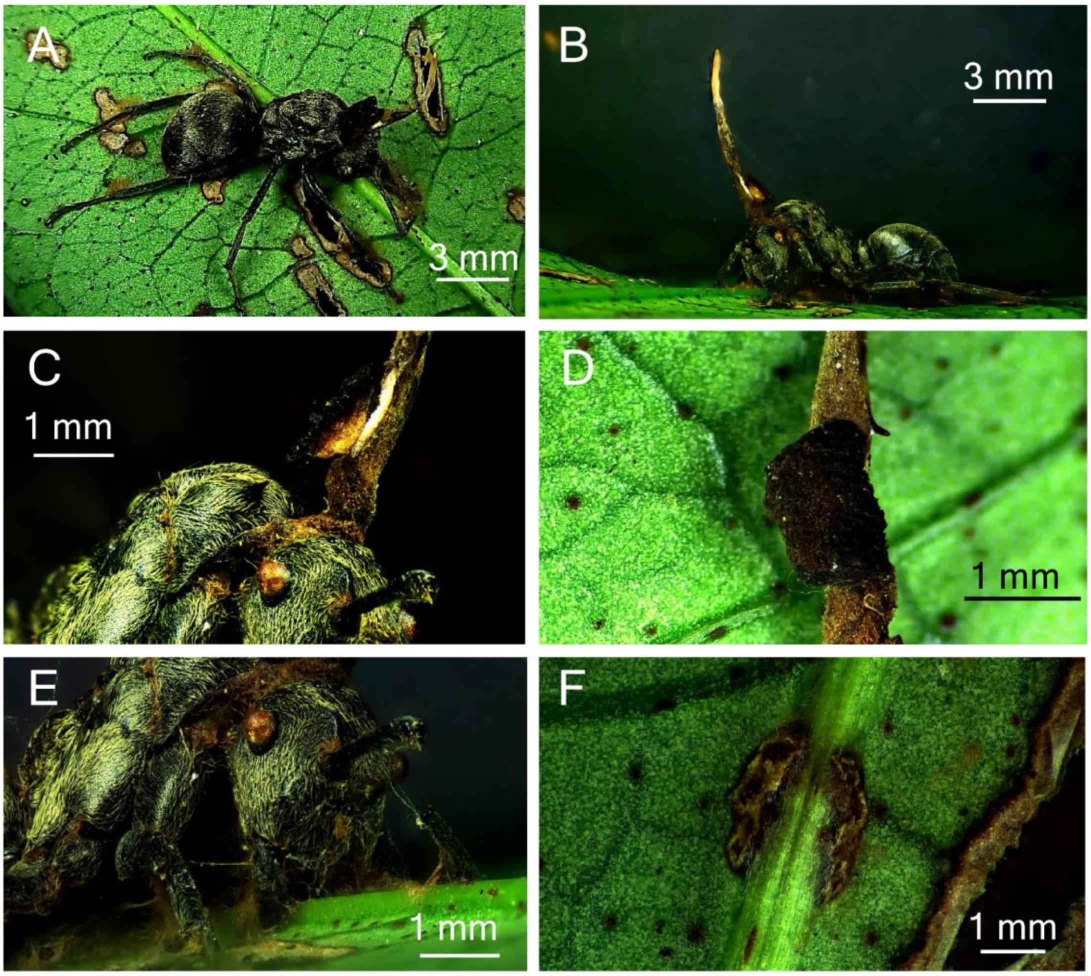

Supplementary Fig. 2 *Ophiocordyceps unilateralis sensu lato*-infected *Polyrhachis wolfi* (A) top view (B) side view (C) emerging stroma from the dorsal neck (D) stromata containing mature perithecia (E) frontal view of the ant biting the vein of the leaf (F) dumbbell-shaped scars centered around its veins after the ant was gently removed from the leaf.

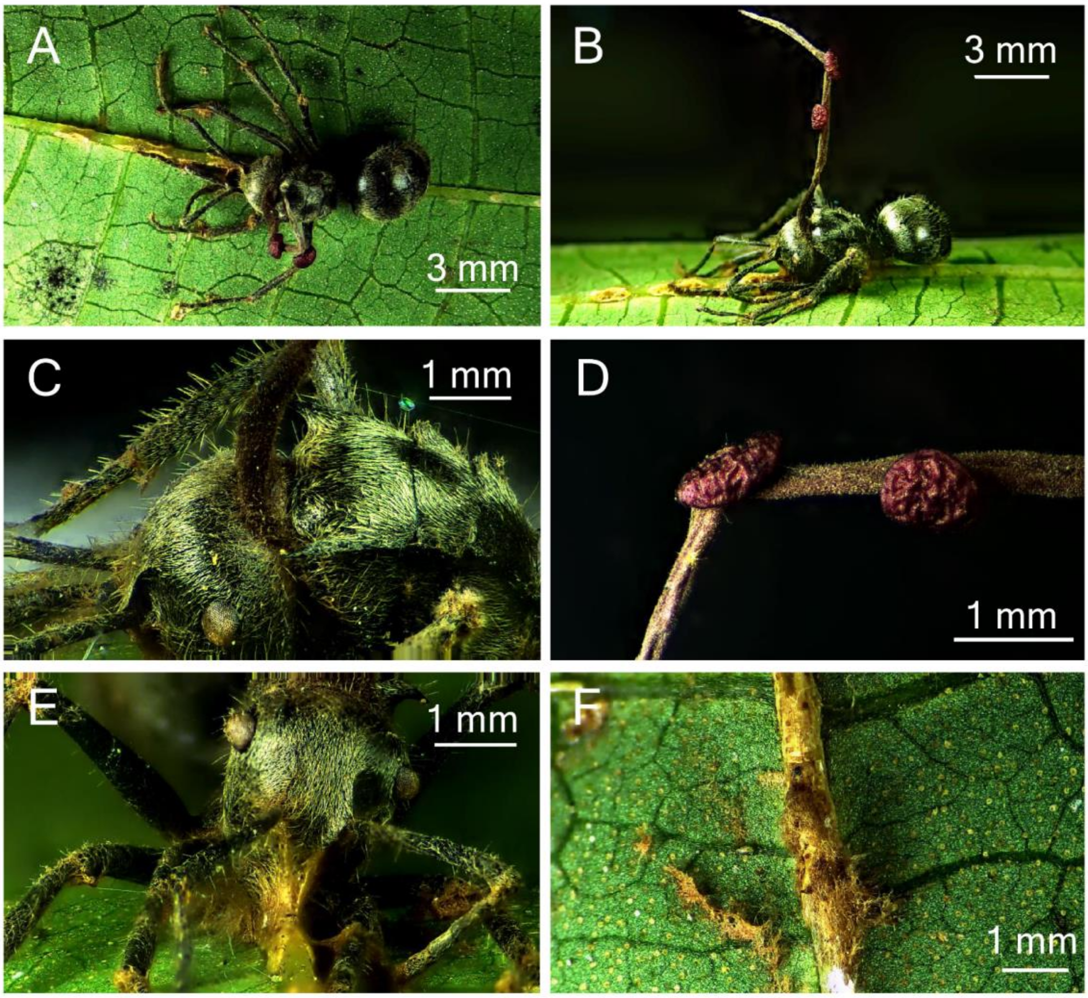

Supplementary Fig. 3 *Ophiocordyceps unilateralis sensu lato*-infected *Polyrhachis vigilans* (A) top view (B) side view (C) emerging stroma from the dorsal neck (D) stromata containing two immature perithecia (E) frontal view of the ant biting the vein of the leaf (F) scars centered around its veins after the ant was gently removed from the leaf.

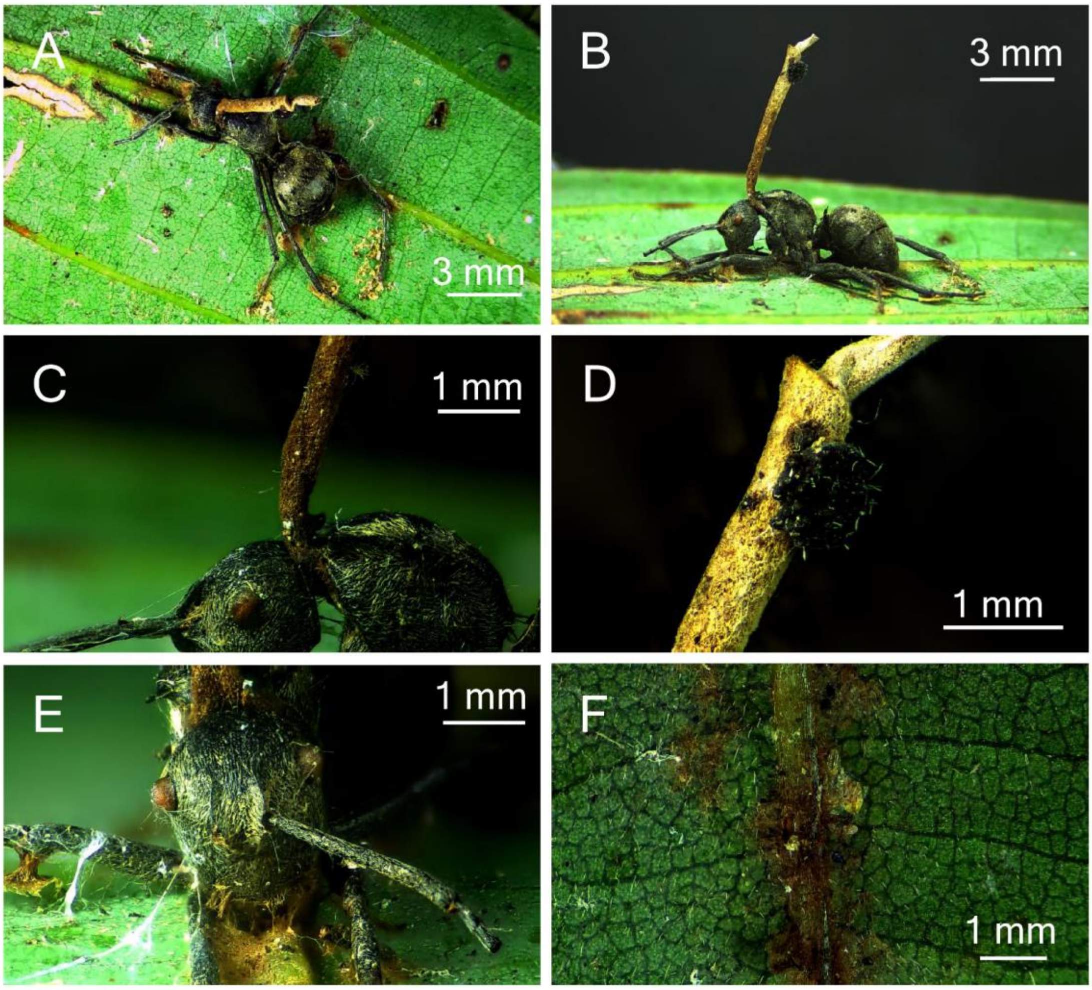

Supplementary Fig. 4 *Ophiocordyceps unilateralis sensu lato*-infected *Polyrhachis latona* (A) top view (B) side view (C) emerging stroma from the dorsal neck (D) stromata containing mature perithecia (E) frontal view of the ant biting the vein of the leaf (F) scars centered around its veins after the ant was gently removed from the leaf.

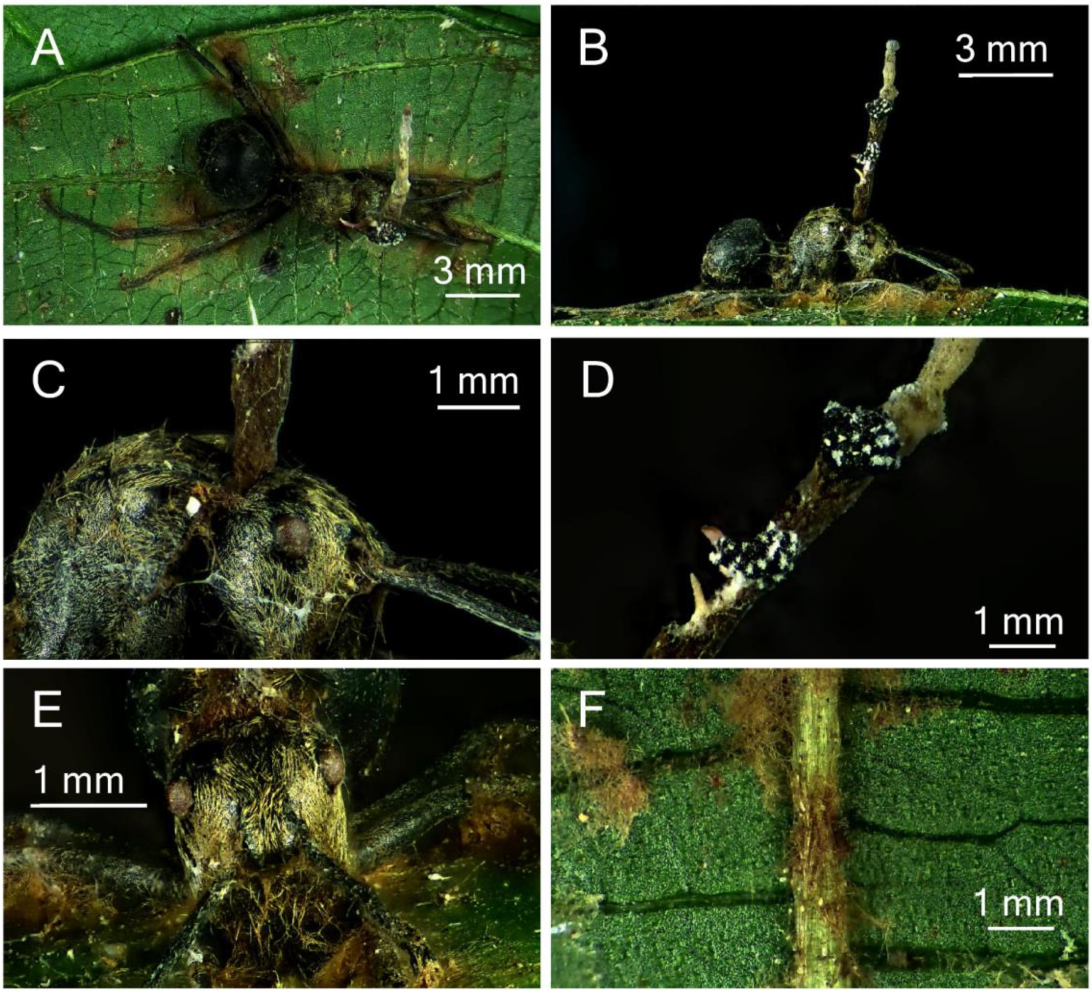

Supplementary Fig. 5 *Ophiocordyceps unilateralis sensu lato* infected *Polyrhachis illaudata* (A) top view (B) side view (C) emerging stroma from the dorsal neck (D) stromata containing two immature perithecia with white hyperparasitic fungi (E) frontal view of the ant biting the vein of the leaf (F) scars centered around its veins after the ant was gently removed from the leaf.

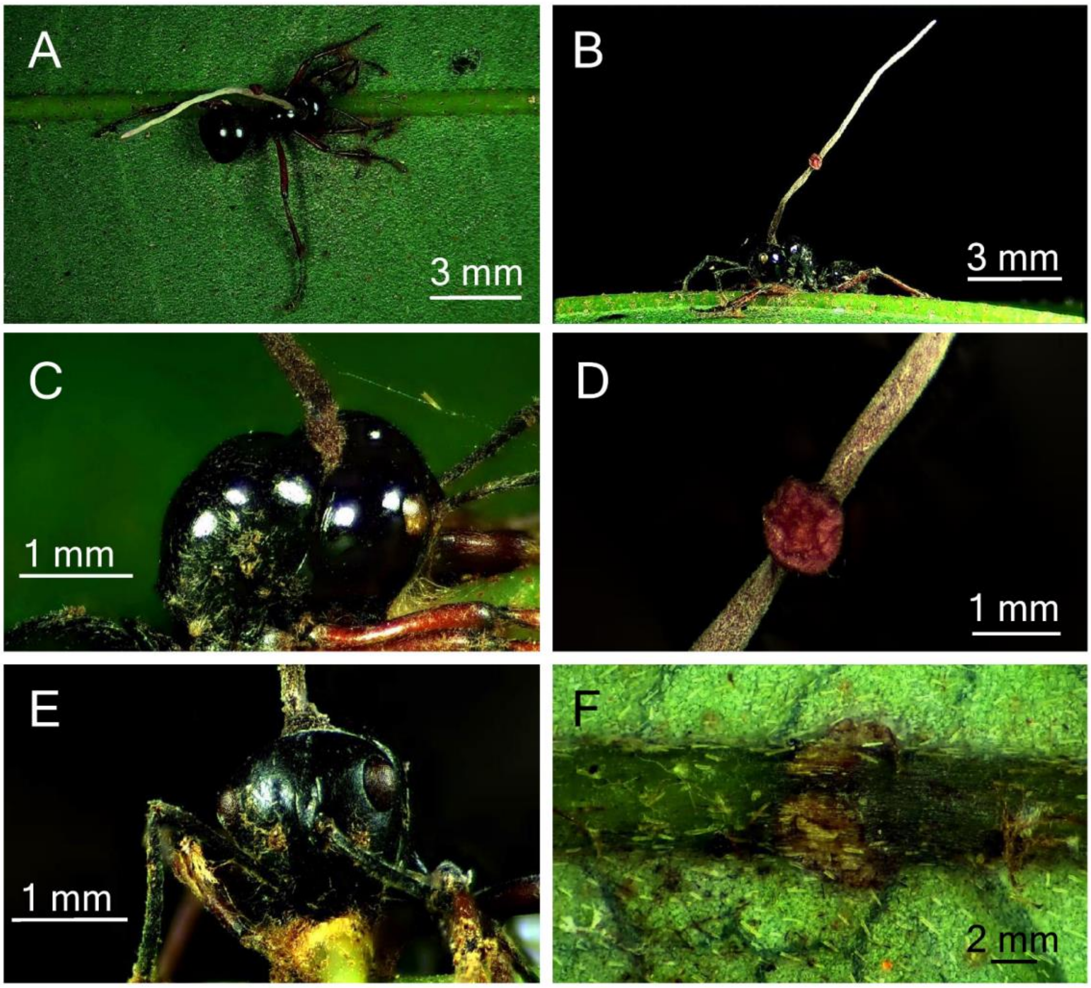

Supplementary Fig. 6 *Ophiocordyceps unilateralis sensu lato*-infected *Polyrhachis debilis* (A) top view (B) side view (C) emerging stroma from the dorsal neck (D) stromata containing immature perithecia (E) frontal view of the ant biting the vein of the leaf (F) dumbbell-shaped scars centered around its veins after the ant was gently removed from the leaf.

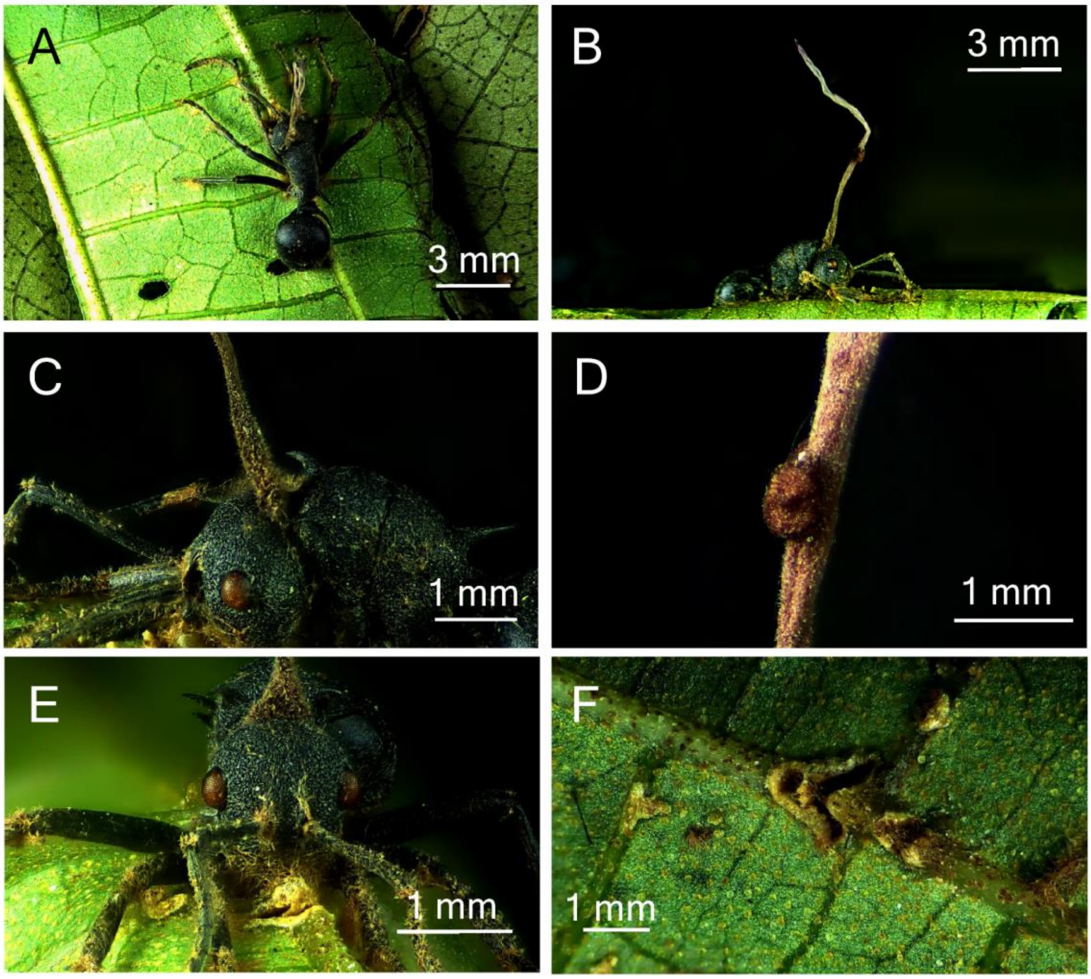

Supplementary Fig. 7 *Ophiocordyceps unilateralis sensu lato*-infected *Polyrhachis dives* (A) top view (B) side view (C) emerging stroma from the dorsal neck (D) stromata containing immature perithecia (E) frontal view of the ant biting the vein of the leaf (F) scars centered around its veins after the ant was gently removed from the leaf.

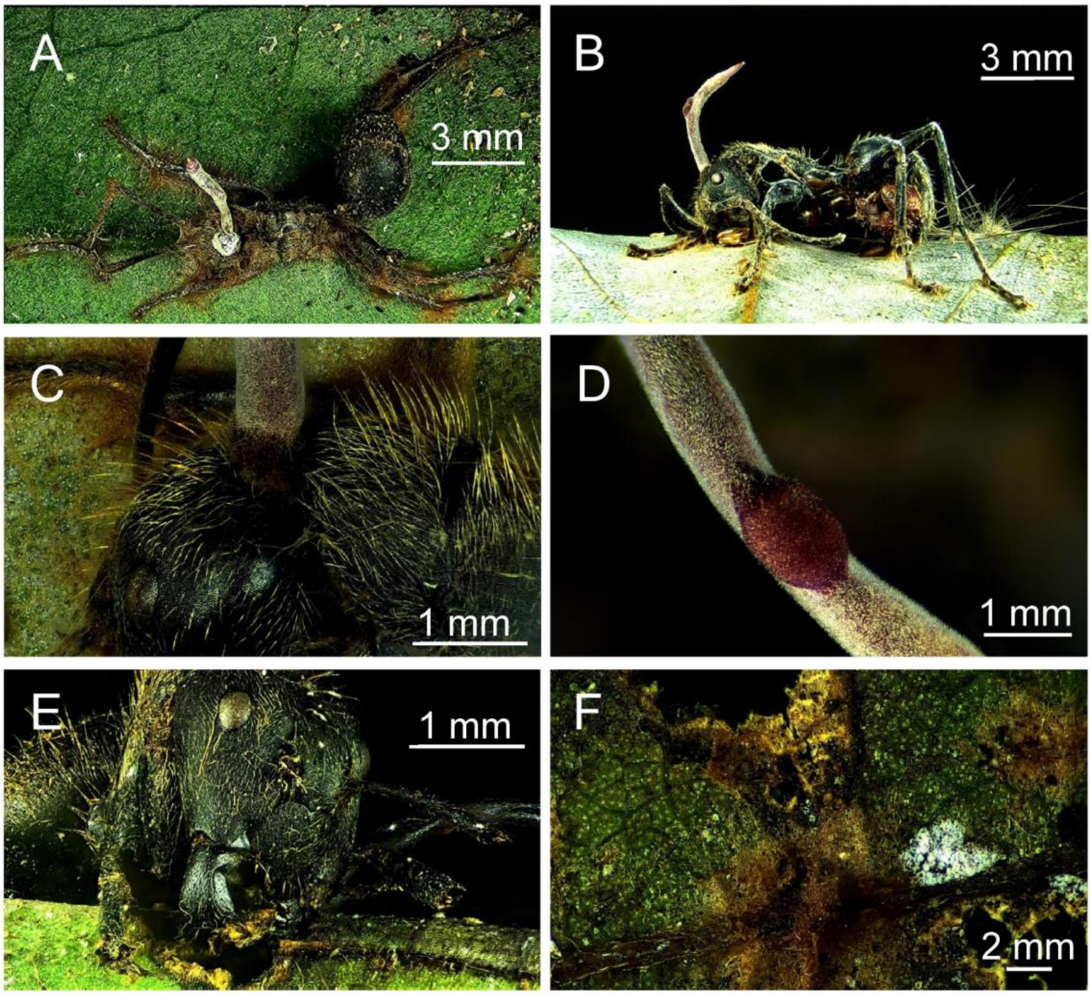

Supplementary Fig. 8 *Ophiocordyceps unilateralis sensu lato*-infected *Camponotus punctatissimus* (A) top view (B) side view (C) emerging stroma from the dorsal neck (D) stromata containing immature perithecia (E) frontal view of the ant biting the vein of the leaf (F) scars centered around its veins after the ant was gently removed from the leaf.

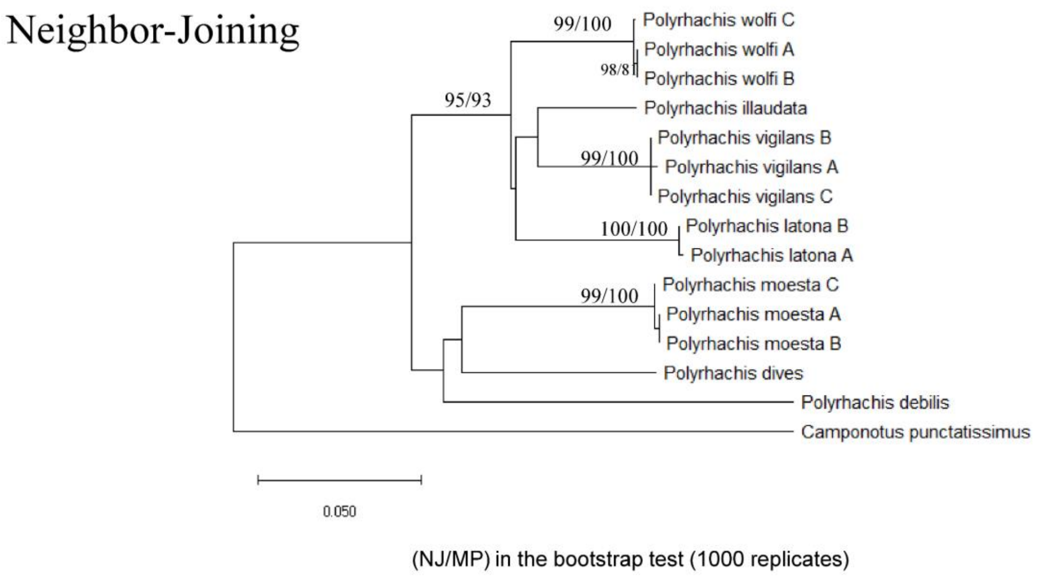

Supplementary Fig. 9 Neighbor-joining *CO2* phylogeny showing that *Ophiocordyceps unilateralis* samples collected in this study infected eight sympatric ant hosts. The statistical support (>50%) based on bootstrap analyses are shown above the branches.

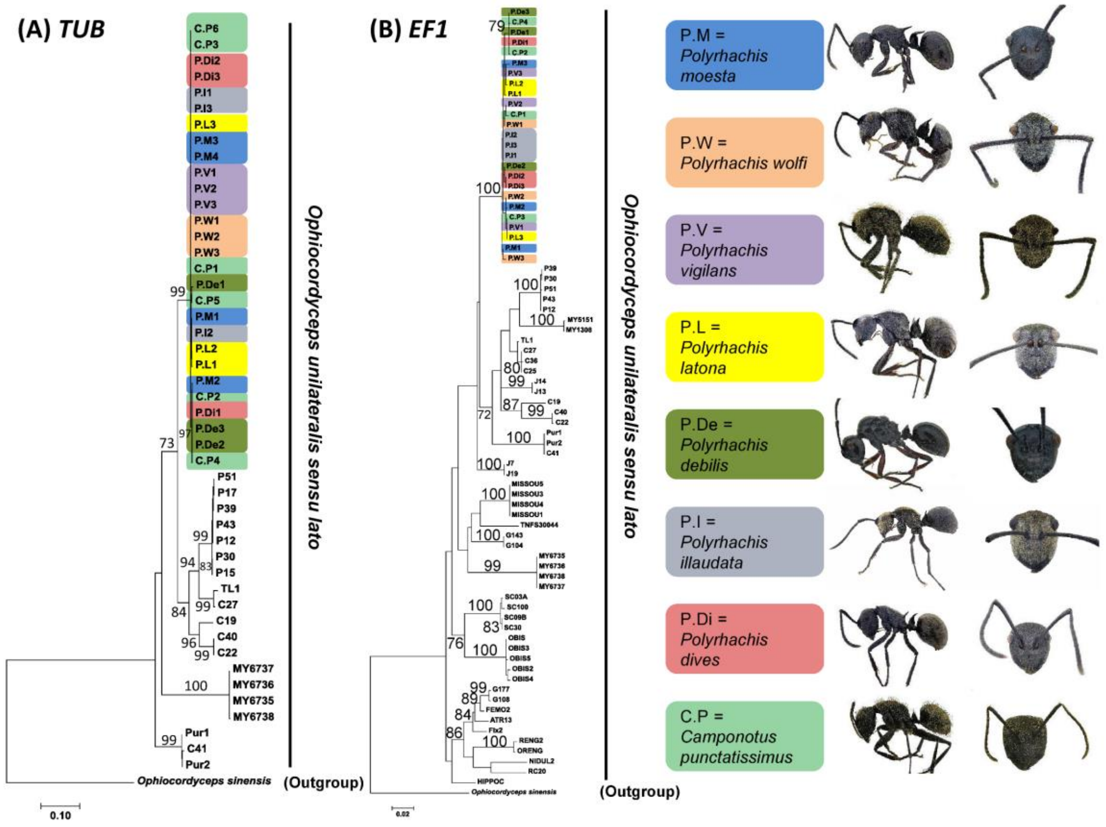

Supplementary Fig. 10 (a) Maximum-likelihood TUB and (b) EF1 phylogeny showing that the infection of different *Ophiocordyceps unilateralis* samples from eight sympatric ant hosts (marked with color) is a monophyletic clade, indicative of the generalized infection of this fungal species. The statistical support (>70%) based on bootstrap analyses are shown above or below the branches. Scale bar indicates substitutions per site.

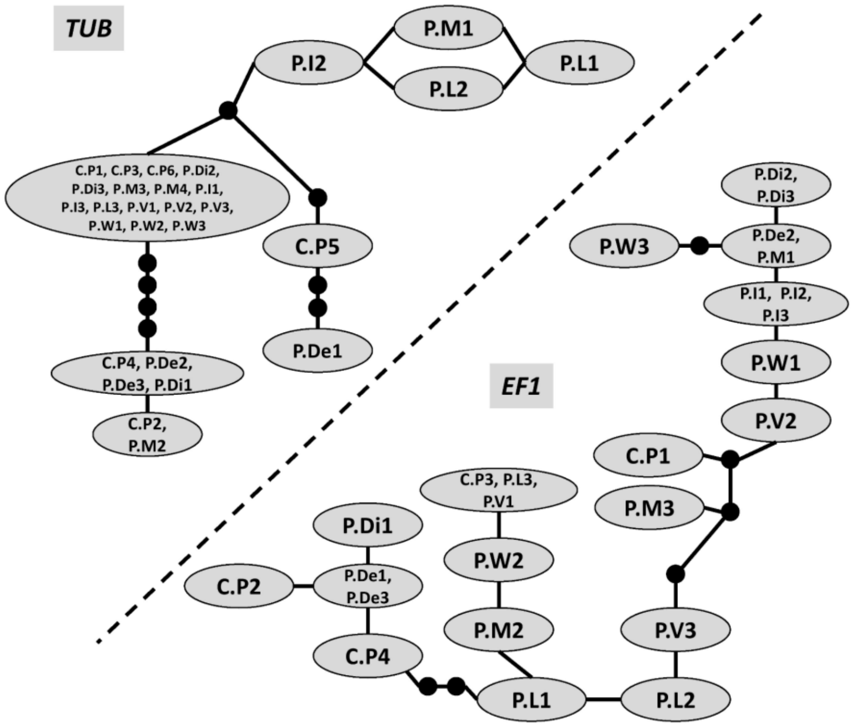

Supplementary Fig. 11 Statistical parsimony network among different haplotypes of TUB (nine in total) and EF1 (18 haplotypes in total). There is no discernible population differentiation from the network analyses among haplotypes of TUB and EF1. Each line between circles represents one mutation step.

Table S1、 List of taxa and fungal samples used in this study, their host identity and corresponding NCBI accession number for EF1 and β-tubulin sequences.

Taxa (as shown on phylogenetic trees)	Species	Host	EF1	TUB
P.M1	*Ophiocordyceps unilateralis sensu lato* [1]	*Polyrhachis moesta*	MN218244	MN218271
P.M2			MN218245	MN218272
P.M3			MN218246	MN218273
P.M4			–	MN218274
P.W1		*Polyrhachis wolfi*	MN218247	MN218275
P.W2			MN218248	MN218276
P.W3			MN218249	MN218277
P.V1		*Polyrhachis vigilans*	MN218253	MN218281
P.V2			MN218254	MN218282
P.V3			MN218255	MN218283
P.L1		*Polyrhachis latona*	MN218250	MN218278
P.L2			MN218251	MN218279
P.L3			MN218252	MN218280
P.De1		*Polyrhachis debilis*	MN218259	MN218287
P.De2			MN218260	MN218288
P.De3			MN218261	MN218289

P.I1		*Polyrhachis illaudata*	MN218256	MN218284
P.I2			MN218257	MN218285
P.I3			MN218258	MN218286
P.Di1		*Polyrhachis dives*	MN218262	MN218290
P.Di2			MN218263	MN218291
P.Di3			MN218264	MN218292
C.P1		*Camponotus punctatissimus*	MN218240	MN218265
C.P2			MN218241	MN218266
C.P3			MN218242	MN218267
C.P4			MN218243	MN218268
C.P5			–	MN218269
C.P6			–	MN218270
P39	*O. polyrhachis-furcata* [2]	*Polyrhachis furcata*	JN819003	JN819051
P51			JN819000	JN819054
P12			JN819030	JN819055
P15			–	JN819060
P17			–	JN819061
P43			JN819001	JN817053
P30			JN819034	JN819058
C36	*O. camponoti-leonardi* [2,3]	*Camponotus leonardi*	JN819013 [2]	–
C27			JN819019 [2]	JN819038 [2]
C25			JN819029 [2]	–

TL1			KJ201526 [3]	KJ201539 [3]
C19	*O. camponoti-saundersi* [2,]	*Camponotus saundersi*	JN819015	JN819042
C40			JN819012	JN819044
C22			JN819014	JN819043
C41	*O. septa* [2, 3]	*Camponotus sp.*	JN819037 [2]	JN819046 [2]
Pur1			KJ201528 [3]	KJ201540 [3]
Pur2			KJ201529 [3]	KJ201541 [3]
MY6735	*O. rami* [3]	*Camponotus sp.*	KJ201531	KJ201535
MY6736			KJ201532	KJ201536
MY6737			KJ201533	KJ201537
MY6738			KJ201534	KJ201538
MY1308	*O. halabalaensis* [4]	*Camponotus gigas*	GU797109	–
MY5151			GU797110	–
TNF-S 30044	*O. pulvinata* [5]	*Camponotus obscuripes*	GU904209	–
MISSOU5	*O. blakebarnesii* [6]	*Camponotus sp.*	KX713688	–
MISSOU4			KX713685	–
MISSOU3			KX713687	–
MISSOU1			KX713686	–
ATRI3	*O. camponoti-atricipis* [6]	*Camponotus atriceps*	KX713677	–
G143	*O. camponoti-balzani* [6]	*Camponotus balzani*	KX713690	–
G104			KX713689	–
OBIS5	*O. camponoti-bispinosi* [6]	*Camponotus bispinosus*	KX713693	–

OBIS4			KX713692	–
OBIS3			KX713695	–
OBIS			KX713694	–
OBIS2			KX713691	–
FEMO2	*O. camponoti-femorati* [6]	*Camponotus femoratus*	KX713678	–
Flx2	*O. camponoti-floridani* [6]	*Camponotus floridanus*	KX713674	–
HIPPOC	*O. camponoti-hippocrepidis* [6]	*Camponotus hippocrepis*	KX713673	–
NIDUL2	*O. camponoti-nidulantis* [6]	*Camponotus nidulans*	KX713669	–
RENG2	*O. camponoti-renggeri* [6]	*Camponotus renggeri*	KX713672	–
ORENG			KX713671	–
G177	*O. camponoti-rufipedis* [6]	*Camponotus rufipes*	KX713680	–
G108			KX713679	–
J19	*O. satoi* [6]	*Polyrhachis lamellidens*	KX713684	–
J7			KX713683	–
SC30	*O. kimflemingiae* [6]	*Camponotus castaneus*	KX713699	–
SC09B			KX713698	–
SC03A			KX713697	–
SC100			KX713696	–
J14	*O. ootakii* [6]	*Polyrhachis moesta*	KX713682	–
J13			KX713681	–
RC20	*O. albacongiuae* [6]	*Camponotus sp.*	*KX713670*	–
Out group	*O.sinensis* [7]	*Lepidopteran pupa*	*JX968018*	*JX968023*

[1] This study

[2] Kobmoo, N., Mongkolsamrit, S., Tasanathai, K., Thanakitpipattana, D., & Luangsa-Ard, J. J. (2012). Molecular phylogenies reveal host-specific divergence of Ophiocordyceps unilateralis sensu lato following its host ants. Molecular Ecology, 21(12), 3022-3031.

[3] Kobmoo, N., Mongkolsamrit, S., Wutikhun, T., Tasanathai, K., Khonsanit, A., Thanakitpipattana, D., & Luangsa-Ard, J. J. (2015). New species of Ophiocordyceps unilateralis, an ubiquitous pathogen of ants from Thailand. Fungal biology, 119(1), 44-52.

[4] Luangsa-Ard, J. J., Ridkaew, R., Tasanathai, K., Thanakitpipattana, D., & Hywel-Jones, N. (2011). Ophiocordyceps halabalaensis: a new species of Ophiocordyceps pathogenic to Camponotus gigas in Hala Bala Wildlife Sanctuary, Southern Thailand. Fungal biology, 115(7), 608-614.

[5] Kepler, R. M., Kaitsu, Y., Tanaka, E., Shimano, S., & Spatafora, J. W. (2011). Ophiocordyceps pulvinata sp. nov., a pathogen with a reduced stroma. Mycoscience, 52(1), 39-47.

[6] Araújo, J. P., Evans, H. C., Kepler, R., & Hughes, D. P. (2018). Zombie-ant fungi across continents: 15 new species and new combinations within Ophiocordyceps. I. Myrmecophilous hirsutelloid species. Studies in Mycology, 90, 119-160.

[7] Zhang, S., Zhang, Y. J., Liu, X. Z., Zhang, H., & Liu, D. S. (2013). On the reliability of DNA sequences of Ophiocordyceps sinensis in public databases. Journal of industrial microbiology & biotechnology, 40(3-4), 365-378.
